# Exploring the HME and HAE1 efflux systems in the genus *Burkholderia*

**DOI:** 10.1186/1471-2148-10-164

**Published:** 2010-06-03

**Authors:** Elena Perrin, Marco Fondi, Maria Cristiana Papaleo, Isabel Maida, Silvia Buroni, Maria Rosalia Pasca, Giovanna Riccardi, Renato Fani

**Affiliations:** 1 Lab. of Molecular and Microbial Evolution, Dep. of Evolutionary Biology, University of Florence, Via Romana 17-19, 50125 Firenze, Italy; 2 Dep. of Genetics and Microbiology, University of Pavia, Via Ferrata 1, 27100 Pavia, Italy

## Abstract

**Background:**

The genus *Burkholderia *includes a variety of species with opportunistic human pathogenic strains, whose increasing global resistance to antibiotics has become a public health problem. In this context a major role could be played by multidrug efflux pumps belonging to Resistance Nodulation Cell-Division (RND) family, which allow bacterial cells to extrude a wide range of different substrates, including antibiotics. This study aims to i) identify *rnd *genes in the 21 available completely sequenced *Burkholderia *genomes, ii) analyze their phylogenetic distribution, iii) define the putative function(s) that RND proteins perform within the *Burkholderia *genus and iv) try tracing the evolutionary history of some of these genes in *Burkholderia*.

**Results:**

BLAST analysis of the 21 *Burkholderia *sequenced genomes, using experimentally characterized *ceoB *sequence (one of the RND family counterpart in the genus *Burkholderia*) as probe, allowed the assembly of a dataset comprising 254 putative RND proteins. An extensive phylogenetic analysis revealed the occurrence of several independent events of gene loss and duplication across the different lineages of the genus *Burkholderia*, leading to notable differences in the number of paralogs between different genomes. A putative substrate [antibiotics (HAE1 proteins)/heavy-metal (HME proteins)] was also assigned to the majority of these proteins. No correlation was found between the ecological niche and the lifestyle of *Burkholderia *strains and the number/type of efflux pumps they possessed, while a relation can be found with genome size and taxonomy. Remarkably, we observed that only HAE1 proteins are mainly responsible for the different number of proteins observed in strains of the same species. Data concerning both the distribution and the phylogenetic analysis of the HAE1 and HME in the *Burkholderia *genus allowed depicting a likely evolutionary model accounting for the evolution and spreading of HME and HAE1 systems in the *Burkholderia *genus.

**Conclusion:**

A complete knowledge of the presence and distribution of RND proteins in *Burkholderia *species was obtained and an evolutionary model was depicted. Data presented in this work may serve as a basis for future experimental tests, focused especially on HAE1 proteins, aimed at the identification of novel targets in antimicrobial therapy against *Burkholderia *species.

## Background

The genus *Burkholderia *is an interesting and complex bacterial taxonomic unit that includes a variety of species inhabiting different ecological niches [[1] and references therein]. In recent years a growing number of *Burkholderia *strains and species have been reported as plant-associated bacteria. Indeed, *Burkholderia *spp. can be free-living in the rhizosphere as well as epiphytic and endophytic, including obligate endosymbionts and phytopathogens. Several strains are known to enhance disease resistance in plants, contribute to better water management, and improve nitrogen fixation and overall host adaptation to environmental stresses [[1] and references therein]. On the other side, some species/isolates can be opportunistic or obligate pathogens causing human, animal or plant disease. Interaction between *Burkholderia *species and humans or animals are traditionally known for *B. mallei *and *B. pseudomallei*, that are the aetiological agent of glanders and melioidosis, respectively [2]. Lastly, several *Burkholderia *species have been demonstrated to be opportunistic pathogens in humans. Although they are not considered pathogens for the normal human population, some are serious threats for specific patient groups. These species include *B. gladioli*, *B. fungorum *and all *B. cepacia *complex (BCC) bacteria [2]. The BCC is a group of genetically distinct but phenotypically similar bacteria that up to now comprises seventeen closely related bacterial species [1,3,4], and they are important opportunistic pathogens that infect the airways of cystic fibrosis (CF) patients [5].

*Burkholderia *human infections are usually treated with antibiotics in order to improve disease control and patient survival. The increasing bacterial resistance to these molecules has become a public health problem. In this context, it seems more and more evident that the intrinsic resistance of many bacteria to antibiotics depends on the constitutive or inducible expression of active efflux systems [6,7]. This is particularly true for multidrug efflux pumps allowing bacterial cells to extrude a wide range of different substrates, including antibiotics. In contrast with other bacterial genes, encoding antibiotic resistance, acquired by horizontal gene transfer (HGT) [8], genes coding for multidrug efflux pumps are mainly harboured by the chromosome(s) of living organisms. In addition, these genes are highly conserved and their expression is tightly regulated [8]. Taken together, these characteristics suggest that the main function of these systems is likely not conferring resistance to antibiotics (used in therapy) and that they might play other roles relevant to the behaviour of bacteria in their natural ecosystems, as also pointed out by Saier and co-workers [9]. According to this idea, it has been recently propose, that MDR proteins might have possessed (and, in some cases, might still possess) a role in preventing the build up of excessive osmotic pressure within the cells, thus functioning as safety valves for normal metabolised substrates [10].

Among the other potential roles, it has been demonstrated that efflux pumps are important for detoxification processes of intracellular metabolites, bacterial virulence in both animal and plant hosts, cell homeostasis and intercellular signal trafficking [8].

**Figure 1:**
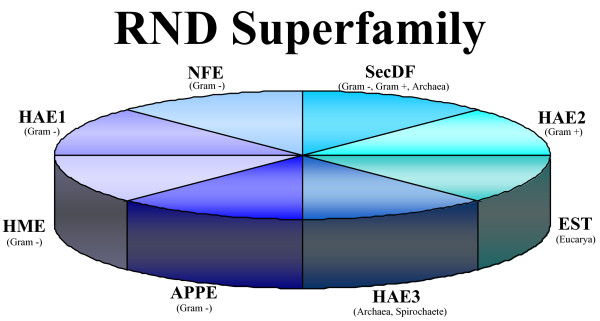
**Schematic representation of the RND superfamily**. APPE = Aryl Polyene Pigment Exporter; EST = Eukaryotic (putative) Sterol transporter; HAE1 = Hydrophobe/Amphiphile Efflux-1; HAE2 = Hydrophobe/Amphiphile Efflux-2; HAE3 = Hydrophobe/Amphiphile Efflux-3; HME = Heavy-Metal Efflux; NFE = putative Nodulation Factor Exporter; SecDF = Secretion system DF family.

This class of proteins includes an ubiquitous and very interesting group, referred to as the RND (Resistance-Nodulation-Cell-Division) superfamily, that is mainly involved in drug resistance of Gram-negative bacteria [11,12]. Functionally characterized members of this superfamily fall into eight different families: four of them are overall restricted to Gram-negative bacteria; the other four families have a diverse phylogenetic distribution (Figure 1). Three of the families peculiar of Gram-negative bacteria have a different substrate specificity, with one catalyzing the export of heavy metals [Heavy Metal Efflux (HME)], one responsible for the export of multiple drugs [Hydrophobe/Amphiphile Efflux-1 (HAE-1)], and the last one likely catalyzing the export of lipooligosaccharides concerned with plant nodulation related to symbiotic nitrogen fixation [putative Nodulation Factor Exporter (NFE)] [13] (Figure 1). The fourth Gram-negative family (APPE) has been only recently identified [14]. It is very distantly related to the other established members of the superfamily and its representatives were shown to be a pigment exporter in *Xanthomonas oryzae *[14].

In Gram-negative bacteria RND transporters act as a complex that can bind various structurally unrelated substrates from the periplasm and/or from the cytoplasm and extrude them out directly into the external media using proton-motive force. This complex is composed of a RND protein, located in the cytoplasmic membrane, a periplasmic-located membrane adaptor protein, belonging to the membrane fusion protein family (MFP), and an outer-membrane channel protein (OMP) [13]. Typically, the encoding genes are organized in an operon and the MFP and the RND are usually cotranscribed [15], whereas in some systems and/or species, the OMP is not linked to the other genes [16,17]. Most of the RND superfamily transport systems consists of a polypeptide chain 700-1300 amino acid residues long. These proteins possess a single transmembrane spanner (TMS) at their N-terminus followed by a large extracytoplasmic domain, six additional TMSs, a second large extracytoplasmic domain, and five final C-terminal TMSs. Most RND permeases consist of a single polypeptide chain [13]. The first half of RND family proteins is homologous to the second one, suggesting that the coding gene is the outcome of an intragenic tandem duplication event of an ancestral gene (i.e. a gene elongation event [18]) that occurred in the primordial system prior to the divergence of the family members [19]. The crystal structure of two tripartite efflux pump components, i.e. the *Escherichia coli *AcrA-AcrB-TolC [20-22] and the *Pseudomonas aeruginosa *MexA-MexB-OprM [23-25] has been determined, whose analysis led to the proposal of a mechanism of drug transport based on the transition through three different conformations [26,27].

Very little is known about RND proteins in the genus *Burkholderia*, whose representatives exhibit multiple antibiotic resistance [28-30]. Indeed, members of RND superfamily have been described for only two species: *B. cenocepacia *and *B. pseudomallei*. In the *B. cenocepacia *J2315 genome, 16 genes encoding putative RND efflux pumps were discovered [31,32]. Two of them have been shown to be associated with drug resistance: i) BCAM2550, *ceoB *(CDS10), a component of a system responsible for chloramphenicol, trimethoprim and ciprofloxacin resistance [33,34]; and ii) BCAS0765 (CDS2) that is associated with resistance to three antibiotics (fluoroquinolones, tetraphenylphosphonium, and streptomycin) as well as to ethidium bromide [31]. In *B. pseudomallei *K96243 at least 10 operons that may code for RND efflux pump components were disclosed [35]. Although differently annotated, these pumps are conserved in other *B. pseudomallei *strains [35]. Three of these systems have been characterized from a functional viewpoint: AmrAB-OprA, BpeAB-OprB and BpeEF-OprC. AmrAB-OprA and BpeAB-OprB are pumps that extrude aminoglycoside and macrolide [36,37], while BpeEF-OprC was shown to efflux trimethoprim and chloramphenicol in a surrogate *P. aeruginosa *strain [38]. Interestingly, the secretion of acyl-homoserine lactones, involved in quorum-sensing systems of *B. pseudomallei*, is absolutely dependent on the function of the BpeAB-OprB [39,40].

Hence, given the clinical/ecological importance of *Burkholderia *representatives, and the importance of RND proteins in antibiotic resistance of Gram-negative bacteria, a large-scale bioinformatic analysis was performed aiming to provide a deeper understanding of RND proteins structure/function in *Burkholderia *genus. The importance of comparative genomics in narrow bacterial groups is an emerging issue [41-43], and it is revealing as a promising approach to gain information about the whole considered clade as well as about its representatives. Such analysis, in fact, can lead to the rapid identification of gene sets that are very likely responsible for the emergence of certain specific phenotypes in a given clade, such as virulence, symbiosis, and so on.

In particular the aims of this work were: 1) to analyze the phylogenetic distribution of CeoB-like pumps in the *Burkholderia *genus; 2) to define the function(s) they perform within the *Burkholderia *genus and 3) to try tracing the evolutionary history of these genes in *Burkholderia*.

## Results and Discussion

### Analysis of the amino acid sequences of the 16 CeoB-like proteins of *B. cenocepacia *J2315

The existence of 16 CeoB-like coding genes in the genome of *B. cenocepacia *J2315 was previously reported [31,32]. However, a deep analysis of these 16 proteins was not carried out until now. To this purpose, each sequence was firstly scanned for the presence of the four highly conserved motifs shared by RND proteins [11,19], whose consensus sequences are shown in Table 1[44]. The analysis of the 16 *B. cenocepacia *J2315 CeoB-like amino acid sequences revealed the existence of the motifs in each of them (see below).

**Table 1:** Consensus sequences of RND proteins according to Putman *at al*. (2000) [44].

MOTIF		**CONSENSUS SEQUENCES**
**A**	**Old**	G x s x v T v x F x x g t D x x x A q v q V q n k L q x A x p x L P x x V q x q g x x v x k
	**Proposed**	G x a x i t x t F x x g t d x d x A x x x V q x x x x x a x x x L P x x v x x p x x x x x x

**B**	**Old**	a l v l s a V F l P m a f f g G x t G x i y r q f s i T x v s A m a l S v x v a l t l t P A l c A
	**Proposed**	t l v l x a V F v P x a f x x G x x G x l f r x f A x t x a x a x x x S x x x a l t L x P a L c a

**C**	**Old**	x x x G k x l x e A x x x a a x x R L R P I L M T s L a f i l G v l P l a i a t G x A G a
	**Proposed**	x x x G x x p x x A x x e A a x l R l R P I l M T x l A x x l G x x P L a x x x G - a G s

**D**	**Old**	S i N t l T l f g l v l a i G L l v D D A I V v V E N v e R v l a e
	**Proposed**	s i N x l s L x g l v L A i G i l V D D A I V v v E N v e R x x x E

Table reported the previously individuated and the new proposed consensus sequence. X indicates any amino acid, capital letters show amino acids most frequently observed in a given position in more than 70% of the transport proteins, and lowercase letters represent amino acid occurring in at least than 40% of RND amino acid sequences.

**Figure 2:**
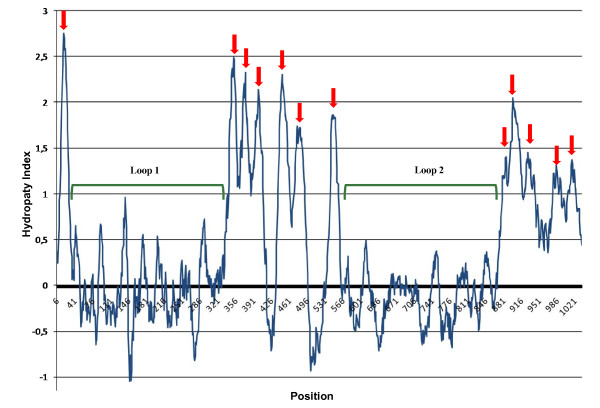
**Averaged hydropathy plot **[**45**]** of *B. cenocepacia *J2315 proteins**. X axis, position on amino acid sequence; y-axis, hydropathy index. Red arrows shows the twelve putative TMS and green bracket the two putative periplasmic loops.

In order to assess the conservation of RND proteins structure of each of the 16 sequences, a hydropathy analysis, using the Kyte and Doolittle hydropathicity scale [45] on ProtScale website http://www.expasy.ch/tools/protscale.html[46] (see Material and Methods), was carried out. The analysis of each of the 16 plots and a comparison with the experimentally determined secondary structure of the *E. coli *AcrB and *P. aeruginosa *MexB (not shown), allowed to identify all the 12 TMS and the two large loops, that are characteristic of RND proteins. An averaged plot of the 16 *B. cenocepacia *J2315 proteins is reported in Figure 2.

### Organization and phylogenetic analysis of *rnd *genes in *B. cenocepacia *J2315

**Figure 3:**
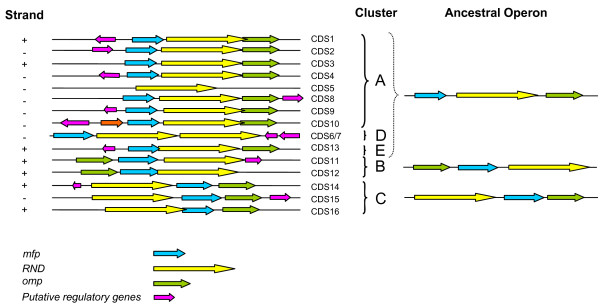
**Schematic representations of the organization of the 16 gene clusters encoding CeoB-like efflux pumps in *B. cenocepacia *J2315 genome**. The organization of the genes identified in *B. cenocepacia *J2315 genome was retrieved from NCBI website http://www.ncbi.nlm.nih.gov/genomes/lproks.cgi. RND transporter-encoding genes are depicted as yellow arrows (CDS1-16), outer-membrane protein-encoding genes as green arrows, periplasmatic membrane fusion protein-encoding genes as pale blue, and putative regulatory genes (physically linked) as pink arrows. *llpe *gene present only in CeoB operon (CDS10) is depicted as orange arrows. Abbrevations: CDS1 (BCAS0592, gi:197295433), CDS2 (BCAS0765, gi:197265565); CDS3 (BCAL1675, gi:206560037); CDS4 (BCAL2821, gi:206561158); CDS5 (BCAL1778, gi:206560142); CDS6 (BCAL1079, gi:206559466)/CDS7 (BCAL1080, gi:206559467); CDS8 (BCAM0926, gi:206562781); CDS9 (BCAM1946, gi:206563791); CDS10 (BCAM2550, gi:206564391); CDS11 (BCAM0713, gi:206562573); CDS12 (BCAM0435, gi:206562298); CDS13 (BCAL1812, gi:206560174); CDS14 (BCAS0582, gi:197295425); CDS15 (BCAM1421, gi:206563273); CDS16 (BCAL2134, gi:206560497).

The analysis of the organization of the 16 *B. cenocepacia *J2315 *rnd *genes (Figure 3) revealed that in most cases the three genes are organized in a putative operon with three different gene arrays:

i) in the first one, shared by CDSs 1-4, 8-10 and 13, the *ceoB *gene is located in between two genes encoding MFP and OMP;

ii) in the second array, shared by CDS11 and 12, the *ceoB *gene is located downstream from the other two genes;

iii) lastly, in the third one, *ceoB *is located upstream of the other two genes.

Besides, in one case (CDS5), the *ceoB-*like sequence is not embedded in a cluster including the other two genes; in another case (CDS6-7), two *ceoB*-like redundant copies were tandemly arranged.

**Figure 4:**
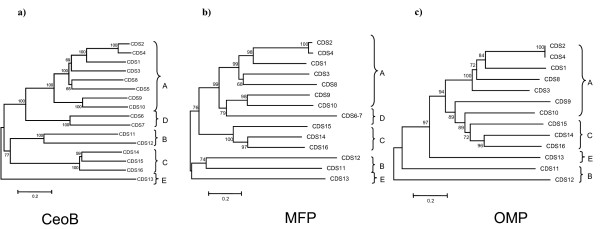
***B. cenocepacia *J2315 CeoB-like sequences phylogenetic tree (a), their MFP associated proteins (b) and OMP proteins (c)**. Abbrevations: a) CDS1 (BCAS0592, gi:197295433), CDS2 (BCAS0765, gi:197265565); CDS3 (BCAL1675, gi:206560037); CDS4 (BCAL2821, gi:206561158); CDS5 (BCAL1778, gi:206560142); CDS6 (BCAL1079, gi:206559466)/CDS7 (BCAL1080, gi:206559467); CDS8 (BCAM0926, gi:206562781); CDS9 (BCAM1946, gi:206563791); CDS10 (BCAM2550, gi:206564391); CDS11 (BCAM0713, gi:206562573); CDS12 (BCAM0435, gi:206562298); CDS13 (BCAL1812, gi:206560174); CDS14 (BCAS0582, gi:197295425); CDS15 (BCAM1421, gi:206563273); CDS16(BCAL2134, gi:206560497) b) CDS1 (BCAS 0591, gi:197295432); CDS2 (BCAS0766, gi:197295596); CDS3 (BCAL1674, gi:206560036); CDS4 (BCAL2822, gi:206561159); CDS6-7 (BCAL1081, gi:206559468); CDS8 (BCAM0927, gi:206562782); CDS9 (BCAM1947, gi:206563792); CDS10 (BCAM2551, gi:206564392); CDS11 (BCAM0712, gi:206562572); CDS12 (BCAM0434, gi:206562297); CDS13 (BCAL1811, gi:206560173); CDS14 (BCAS0583, gi:197295426); CDS15 (BCAM1420, gi: 206563272); CDS16 (BCAL2135, gi:206560498). c) CDS1 (BCAS0593, gi:197295434); CDS2 (BCAS0764, gi:197295594); CDS3 (BCAL1676, gi:206560038); CDS4 (BCAL2820, gi:206561157); CDS8 (BCAM0925, gi:206562780); CDS9 (BCAM1945, gi:206563790); CDS10 (BCAM2543, gi:206564390); CDS11 (BCAM0711, gi:206562571); CDS12 (BCAM0433, gi:206562296); CDS13 (BCAL18513, gi:206560175); CDS14 (BCAS0584, gi:197295427); CDS15 (BCAM1419, gi:206563271); CDS16 (BCAL2136, gi:206560499).

In order to analyse the phylogenetic relationships among the 16 CeoB-like proteins their amino acid sequences were aligned using the program ClustalW [47] and the multialignments obtained were used to construct the phylogenetic tree shown in Figure 4a. The topology of the tree, which is supported in most cases by very high bootstrap values, revealed that the 16 sequences can be split into five clusters (A, B, C, D, and E).

It is worth noting that the overall different gene organization of CeoB, MFP and OMP coding genes is consistent with the subdivisions in the phylogenetic tree in Figure 4a.

A similar phylogenetic analysis was also performed using the aminoacid sequence of MFP and OMP proteins encoded by genes embedding each operon. Data obtained revealed that the five clusters (A, B, C, D, and E) can be easily recognized in the MFP tree (even though the branching order is different) (Figure 4b). This is in agreement with the notion that the two proteins have to interact and often belong to the same transcriptional unit. The topology of OMP tree is slightly different from the other two, in that some of the sequences of cluster A are intermixed with those of cluster C (Figure 4c). This finding might suggest that this gene could have followed a (partially) independent evolutionary pathway, which is also in agreement with the fact that in some cases it is missing in the RND operons.

### Identification and distribution of *ceoB*-like genes in the genus *Burkholderia*

In order to check the distribution of the CeoB-like proteins in the entire genus *Burkholderia*, the *B. cenocepacia *J2315 CeoB amino acid sequence (gi:206564391) was used as a query to probe the 21 completely sequenced genomes of strains belonging to *Burkholderia *genus available at NCBI database http://www.ncbi.nlm.nih.gov (1/05/2009), using default parameters. In this way, a total of 254 sequences homologous to *B. cenocepacia *J2315 CeoB were retrieved.

**Figure 5:**
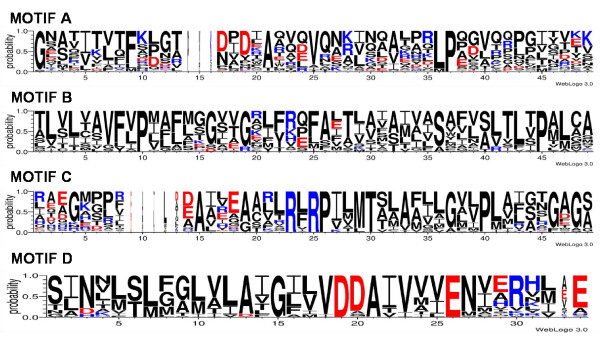
**WebLogo representation of the four highly conserved motifs shared by *Burkholderia *RND proteins**. Amino acids with a positive charge are represented in blue; amino acids with a negative charge are represented in red; amino acids without any charge are represented in black.

Each sequence was analyzed for the presence of the four highly conserved motifs shared by RND proteins [11,19]. Data obtained (not reported) revealed the existence of the four motifs in all the 254 *Burkholderia *sequences, supporting the idea that they actually are members of RND superfamily. The relative frequency of each amino acid in each position was checked using the WebLogo application (see Material and Methods) (Figure 5). In some cases this frequency differed from the consensus sequence(s) previously suggested [44]. This is due to the fact that our dataset includes a larger number of sequences in respect to the previous ones [11,19,48]. Hence, we suggested new possible consensus motifs for these sequences (Table 1).

Figure 5 and Table 1 show that Motif A and Motif D represent the least and the most conserved ones, respectively. This is in agreement with the notion that Motif A is located on the first periplasmatic loop; many studies demonstrated that periplasmatic regions of RND proteins are involved in substrate recognition [49-54]. Thus, a higher sequence variability of Motif A among various proteins is consistent with the possible recognition of different substrates. On the other hand, part of Motif D coincides with TMS4, which is involved in proton translocation [55,56], a function common to all RND proteins. Thus, as expected, this region should exhibit a high degree of conservation among proteins transporting different substrates.

As shown in Table 2, a highly variable number of CeoB-like proteins, ranging from 6 (in *B. mallei *NCTC10247, NCTC10229 and SAVP1) to 18 (in *B. cenocepacia *HI2424 and MC0-3) was found.

**Table 2:** Total number of RND proteins and number of RND proteins of each type present in each family of the 21 *Burkholderia *analysed genomes.

	Number of												
	
Species	CeoB-like Proteins	HAE1 (A)	HAE1 (D)	HAE1 (E)	tot HAE1	CDS 11 (B)	CDS 12 (B)	tot HME	CDS 14 (C)	CDS 15 (C)	CDS 16 (C)	tot UF	NC
*B. ambifaria*	11	3	2	2	7	-	1	1	1	1	1	3	-
	13	4	2	3	9	-	1	1	1	1	1	3	-
*B. cenocepacia*	18	9	2	2	13	1	1	2	1	1	1	3	-
	17	8	2	2	12	1	1	2	1	1	1	3	-
	16	8	2	1	11	1	1	2	1	1	1	3	-
	18	9	2	2	13	1	1	2	1	1	1	3	-
*B. lata*	13	8	2	1	11	1	-	1	-	1	-	1	-
*B. mallei*	6	3	1	-	4	1	1	2	-	-	-	-	-
	6	3	1	-	4	1	1	2	-	-	-	-	-
	6	3	1	-	4	1	1	2	-	-	-	-	-
	7	3	1	1	5	1	1	2	-	-	-	-	-
*B. multivorans*	12	5	2	1	8	1	2	3	-	1	-	1	-
*B. phymatum*	16	7	4	3	14	-	-	-	1	1	-	2	-
*B. phytofirmans*	16	7	4	1	12	-	-	-	2	1	-	3	1
*B. pseudomallei*	10	4	3	1	8	1	1	2	-	-	-	-	-
	10	4	3	1	8	1	1	2	-	-	-	-	-
	10	4	3	1	8	1	1	2	-	-	-	-	-
	10	4	3	1	8	1	1	2	-	-	-	-	-
*B. thailandensis*	11	4	3	1	8	1	-	1	-	-	-	-	2
*B. vietnamiensis*	11	5	2	1	8	-	1	1	1	1	-	2	-
*B. xenovorans*	17	6	4	1	11	1	-	1	2	1	2	5	-

Abbreviations: HAE1: Hydrophobe/Amphiphile Efflux-1, HME: Heavy-Metal Efflux, UF: Uncertain Function, NC: Not Classified

**Figure 6:**
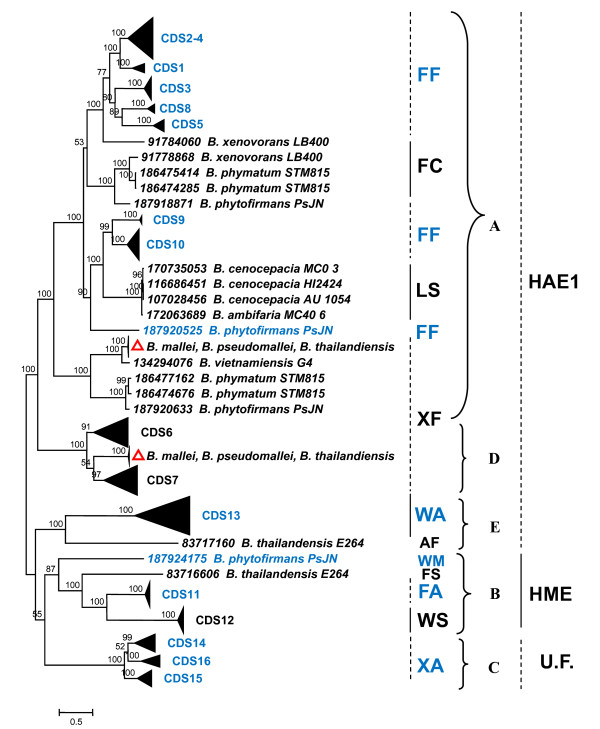
**Schematic representation of phylogenetic tree constructed using the 254 *Burkholderia *CeoB-like sequence**. Sequences that present the same residues in positions corresponding to positions 4-5 of *E. coli *AcrB are highlighted. Abbreviations: A = Alanine; C = Cysteine; F = Phenylalanine; L = Leucine; M = Methionine; S = Serine; W = Tryptophan; X = any hydrophobic aminoacid; HAE1 = Hydrophobe/Amphiphile Efflux-1; HME = Heavy-Metal Efflux; U.F. = Uncertain Function.

The 254 *Burkholderia *amino acid sequences retrieved were then aligned using the Muscle program (see Material and Methods); the obtained multialignment was then used to construct the phylogenetic tree schematically reported in Figure 6 (the entire tree is available in Additional File 1). In the same Figure residues characterising each group of sequences are also reported (see following sections). The analysis of phylogenetic tree revealed that the majority of sequences form clusters including one of the *B. cenocepacia *J2315 sequences (Figure 6, black triangles), while other sequences form clusters that do not comprise any *B. cenocepacia *J2315 sequence. In particular, two of these clusters contain sequences from only *B. mallei*, *B. pseudomallei *and *B. thailandiensis *(highlighted with red triangles in Figure 6). However, in the whole phylogenetic tree, the sequences can be easily subdivided into the five clusters (A, B, C, D, and E) corresponding to the previously identified ones (Figure 3a) (although embedding a variable number of sequences).

### Functional assignment of the 254 *Burkholderia *CeoB-like sequences

A preliminary analysis, performed by aligning all the 254 *Burkholderia *sequences with the sequences representative of the five RND families identified in Gram-negative bacteria and experimentally characterized retrieved from Transport Classification Database (TCDB, http://www.tcdb.org) [57], revealed that most of these sequences could be unambiguously assigned to only two RND families: HAE1 and HME. Indeed, only sequences belonging to these two families shared a significant degree of similarity with the *Burkholderia *sequences, whereas those representative of the other three families (NFE, APPE, SEC DF) resulted highly divergent from the *Burkholderia *ones and could not be reliably aligned (the average degree of identity between the 254 *Burkholderia *sequences and those representative of these families are: 23% for NFE proteins, 11% for APPE family and 9% for SecDF family).

To confirm this preliminary assignment and try to determine the substrate of each pump, three different analyses were performed:

i) comparison of the 254 CeoB-like sequences with the amino acid sequence of HAE1 and HME experimentally characterized proteins, belonging to other microorganisms;

ii) analysis of highly conserved amino acid residues, essential for proton translocation;

iii) analysis of residues involved in substrate recognition.

#### Comparison with HAE1 and HME experimentally characterized proteins belonging to other microorganisms

**Figure 7:**
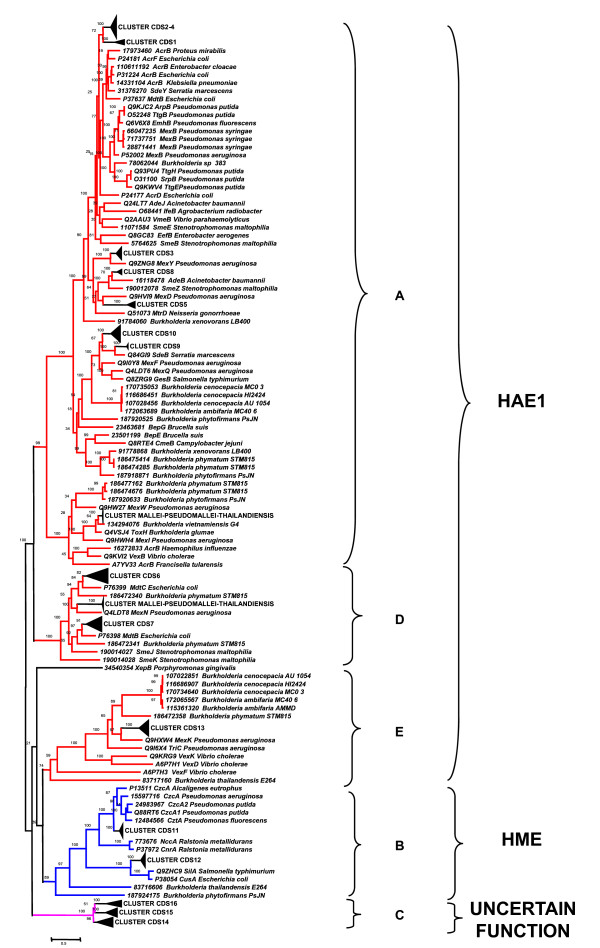
**Schematic representation of the phylogenetic tree constructed using the 254 *Burkholderia *CeoB-like sequences plus sequences of characterized proteins**.

A set of 62 sequences representative of HAE1 and HME families was retrieved from both TCDB and literature (all proteins and their relative substrate are reported in Additional File 2) and aligned with the 254 *Burkholderia *sequences. The multialignment was used to build the phylogenetic tree reported in Additional File 3, and a phylogenetic tree including a subset of these is shown in Figure 7, where the five major clusters (A, B, C, D, E) of Figures 3 and 6 were easily recognized. Three of these clusters (A, D and E, red branches) included characterized proteins belonging to HAE1 family that are known to be involved in antibiotic(s) resistance.

Another cluster (in blue) comprised HME proteins (Cluster B in Figure 6), involved in heavy metal efflux.

Lastly, none of the characterized proteins showed similarity with those grouped in cluster C (pink branches). Indeed, no function could be assigned to these proteins, although they appear to be closer to HME than HAE1 sequences.

The analysis of substrate specificity of each characterized protein revealed that HME proteins, transporting different metals, form two distinct clusters. The first one, contained the protein gi:206562298 from *B. cenocepacia *J2315 (CDS 12), which transports monovalent cations (Cu^+ ^and Ag^+^), the other one, included the sequence gi:206562573 from *B. cenocepacia *J2315 (CDS11), transporting divalent cations (Zn^2+^, Co^2+^, Cd^2+ ^and Ni^2+^).

#### Analysis of highly conserved amino acid residues essential for proton translocation

It has been proposed that some charged residues in TMS 4 and TMS 10 sequences are essential for proton translocation and pumping function of RND proteins [55,56,58]. Some of these residues are highly conserved in all RND proteins, while others are characteristic of HAE1 and HME families.

**Figure 8:**
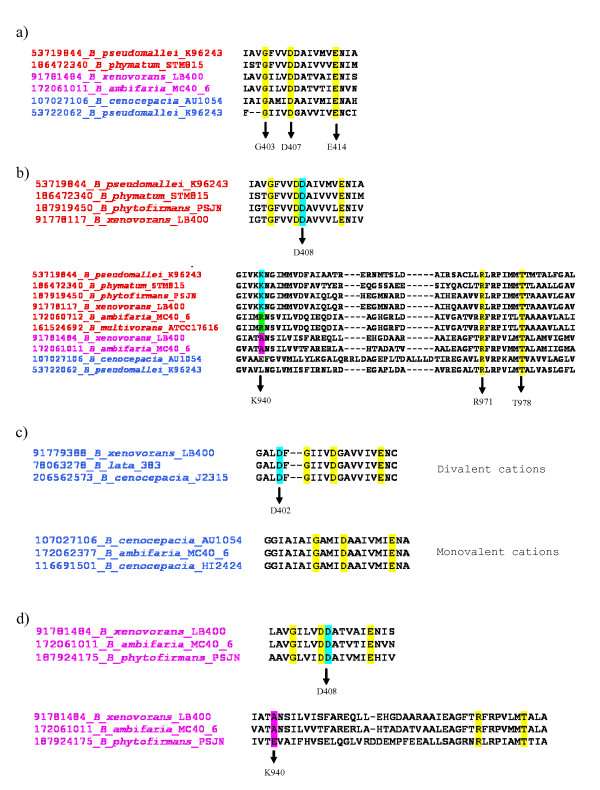
**Essential residues for proton translocation in RND proteins**. Only some representative proteins for each category were reported. Putative HAE1 proteins are coloured in red, putative HME proteins are coloured in blue and proteins with uncertain function in pink. Residues conserved among different proteins are highlighted.

The multiple alignment of the amino acid sequences of 39 proteins, belonging to HAE1 and HME families, revealed that the motif G403XXXD407XXXXXXE414 (position referred to *P. aeruginosa *MexB) in TMS 4 is highly conserved in both HAE1 and HME [56]. This suggests that these residues may play an important role in proton translocation, a feature shared by all the representatives of the families [56]. We checked for the presence of such residues in the 254 *Burkholderia *sequences and all of them were found in each sequence (yellow residues in Figure 8a). This finding confirms that these residues are very likely essential for the role performed by these proteins.

Five residues were conserved in the HAE1 family [56,58]. Two of them, D407-D408 (position referred to *E. coli *AcrB), are located in TMS 4; the other three residues, K940, R971 and T978 (position referred to *E. coli *AcrB), are within or close to TMS 10. As shown in Figure 8b, R971 and T978 are conserved in all sequences, suggesting that they may play an important role for both HAE1 and HME proteins. D407 and D408 are conserved, with the exception of four sequences, in all the proteins that, on the basis of phylogenetic tree, were assigned to the HAE1 family. K940 is conserved in all putative HAE1 sequences, with the exception of one cluster (containing CDS 13 from *B.cenocepacia *J2315), where Lysine is replaced by Arginine. However, mutation study in *E. coli *[56] suggested that in this particular position the side-chain length is not important, and a positive charge is "simply" required.

HME proteins, transporting divalent cations, e.g. *Ralstonia metallidurans *CzcA, possess an Aspartic acid residue at position 402 in TMS 4 (position referred to CzcA of *R. metallidurans*), in addition to previously identified D407 (that, in this kind of proteins, is located at position 408). HME proteins that transport monovalent cations, e.g. *E. coli *CusA, present only D408 and miss D402, which may be explained by a 1H^+^/Ag^+ ^ratio of transport by this system in contrast with a ratio of 2 H^+^/1 Zn^2+ ^for CzcA-like proteins [55]. Figure 8c shows that proteins previously identified as divalent cation transporters, harbour both Aspartic acid residues, whereas proteins identified as monovalent cations transporters, contain only one Aspartic acid (D408).

Lastly, the sequences with unknown function, present the same residues of HAE1 proteins in TMS 4, but miss K940 that is conserved in all HAE1 proteins (Figure 8d).

Thus, the analysis of functional residues of RND proteins confirms that sequences identified in *Burkholderia *spp. are RND proteins, and this is in agreement with phylogenetic analysis data. Indeed, putative HAE1 and HME proteins present residues characteristic of each family, and proteins with uncertain function confirm their apparent ambiguous collocation.

#### Analysis of residues involved in substrate recognition

The analysis of the amino acid sequences of *E. coli *AcrB (HAE1) and its homologs allowed to identify conserved residues at their N-terminus, including two Phenylalanine residues (FF, positions 4-5 of *E. coli *AcrB) exposed to the cytoplasm [59]. Since Phenylalanine residues located elsewhere in the protein sequence have been postulated to be involved in ligand binding, *Das et al. *suggested that these conserved residues might be involved in cytoplasmic substrate recognition [59].

The analysis of the residues located at these positions in the 254 *Burkholderia *sequences revealed that different clusters exhibited different residues (Figure 6):

• a large cluster of proteins (Cluster A) shows (with the exceptions of some sequences) two Phenylalanines (FF) at both positions;

• another cluster (Cluster D), previously identified as HAE1, presents a hydrophobic amino acid at the first position and a Phenylalanine at the second one (XF);

• the third HAE1 cluster (Cluster E) exhibits a Tryptophan and an Alanine (WA);

• a putative HME cluster for divalent cations (CDS11) (Cluster B) presents a Tryptophan and a Serine (WS);

• a putative HME cluster for monovalent cations (CDS12) (Cluster B) possesses a Phenylalanine and an Alanine (FA);

• the sequence cluster with uncertain function (Cluster C) presents a hydrophobic amino acid at the first position and an Alanine at the second position (XA).

Hence, the whole body of data presented strongly suggests that the 254 *Burkholderia *sequences are representative of HAE1 and HME families. In particular, HAE1 proteins can be split into three different groups that likely transport different substrates. HME proteins are divided into two different clusters, one for monovalent and one for divalent cation export, respectively. The third protein cluster cannot be assigned to any of the two families.

### Interrelationships between number and/or type of CeoB-like proteins and genome size, lifestyle, pathogenicity and taxonomic position

The number of CeoB-like proteins of each *Burkholderia *strain was correlated to the genome size, the lifestyle, the pathogenicity and the taxonomic position in order to assess the presence of some (possible) interrelationships. Data of genome size, lifestyle and pathogenicity were retrieved from NCBI website http://www.ncbi.nlm.nih.gov/genomes/lproks.cgi, (Table 3).

**Table 3:** Genome, genome size, habitat and pathogenicity of the 21 *Burkholderia *analysed genomes.

Species	Strain	Habitat	Pathogenicity	Genome size (Mpb)	Chromosomes	Plasmids
*B. ambifaria*	AMMD	E/H	NP	7,57	3	1
	MC40-6	E/H	P	7,60	3	1
*B. cenocepacia*	HI2424	E/H	P	7,76	3	1
	AU 1054	H	P	7,28	3	-
	J2315	E/H	P	8,07	3	1
	MC0-3	E/H	P	7,90	3	-
*B. lata*	383	E/H	P	8,69	3	-
*B. mallei*	NCTC 10247	H	P	5,90	2	-
	NCTC 10229	H	P	5,80	2	-
	SAVP 1	H	P	5,20	2	-
	ATCC 23344	H	P	5,83	2	-
*B. multivorans*	ATCC 17616	H	P	6,99	3	1
*B. phymatum*	STM815	H	NP	8,70	2	2
*B. phytofirmans*	PsJN	E	NP	8,22	2	1
*B. pseudomallei*	1106a	E	P	7,10	2	-
	668	E	P	7,00	2	-
	1710b	E	P	7,31	2	-
	K96243	E	P	7,30	2	-
*B. thailandensis*	E264	E	NP	6,72	2	-
*B. vietnamiensis*	G4	E/H	P	8,40	3	5
*B. xenovorans*	LB400	E/H	NP	9,80	3	-

Abbreviations: E: environmental; H: host; P: pathogen; NP: Not Pathogen; Mbp: Mega base pair.

Three different categories were considered for lifestyle: strains that live predominantly either in environment (water, soil, rhizosphere etc.), or in a host (plants, animals, humans) and strains that can be found in both environment and host. The average number of proteins in each category is very similar, and standard deviation is very high (2.40 for the first category, 4.93 for the second one and 2.97 for the third one) (Additional File 4). Thus, no apparent relationship between bacterial lifestyle and RND protein number was detected. The same result was obtained considering the number of each type of CeoB-like proteins (HAE1, HME and uncertain function) (Additional File 4).

The relationship with pathogenicity (strains pathogens for plants, animals or humans) was also analysed. Also in this case, no apparent relationship exists with protein number (Additional File 4). The same result was obtained considering the number of each type of CeoB-like proteins (HAE1, HME and uncertain function) (Additional File 4).

**Figure 9:**
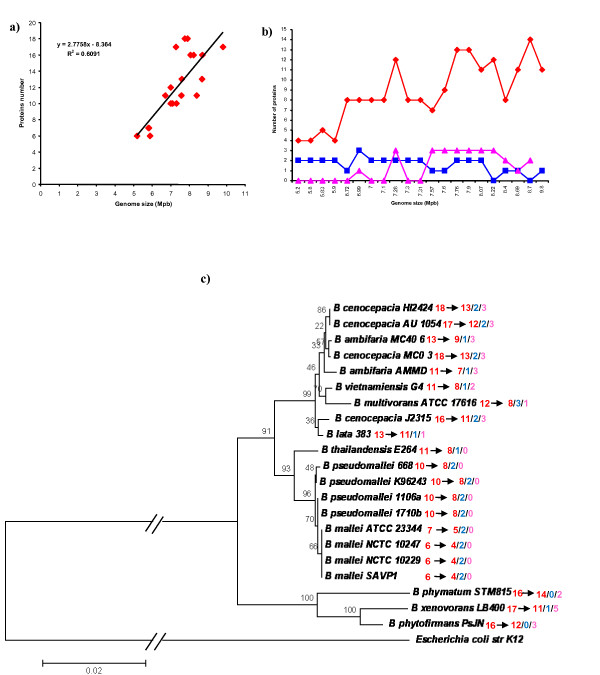
**Relationship among number of CeoB-like proteins and genome size**. (a). Relationship among number of CeoB-like proteins for each type and genome size (b) and taxonomy (c).

In spite of the fact that previous studies suggested that the number of multidrug efflux pumps is proportional to the genome size of a given organism [60], data reported in Figure 9a revealed that in *Burkholderia *genomes only a low correlation between the two parameters exists (R^2 ^= 0.6091). However, when the CeoB-like proteins were split into the three categories, the analysis revealed that the number of HME proteins (blue line, R^2 ^= 0.3787) and of proteins with uncertain function (pink line, R^2 ^= 0.4579), is relatively constant, while the number of HAE1 proteins (red line, R^2 ^= 0.6323) increases in strains with a larger genome (Figure 9b).

In order to assess relationships with taxonomy, the phylogenetic tree reported in Figure 9c was constructed using the 16S rRNA gene sequences of each strain; in this tree, the number of proteins for each strain is also reported. A relationship between number of proteins and taxonomy can be found. Indeed strains of the same species and/or strains of related species, possess an identical or very similar number of RND proteins. Thus, the distribution of CeoB-like proteins belonging to the three identified categories [antibiotic transport (HAE1), heavy metal transport (HME) and uncertain function], coded for by each of the 21 *Burkholderia *genomes, was also analyzed.

Data obtained are summarized in Table 2 and Figure 9c and showing that:

i) proteins with uncertain function are not present in *B. mallei*, *B. pseudomallei *and *B. thailandiensis *strains;

ii) proteins belonging to HME family are not present in *B. phymatum *and *B. phytofirmans*;

iii) when different strains belonging to the same species possess a different number of RND proteins, this is due to the different number of HAE1 proteins, while proteins with uncertain function and HME maintain the same number in all strains of the same species;

iv) HAE1 proteins are the most abundant in all analyzed strains.

### Evolution of *rnd *encoding genes in *Burkholderia *genus

The analysis of the distribution of HME and HAE1 like coding sequences in the genus *Burkholderia *revealed a high variability in the copy number among the different species (Table 2). Interestingly, all the species branching at the root of the *Burkholderia *reference tree (as assessed by 16S rRNA coding sequences), possess a high number of HME/HAE1-like coding sequences (16 in *B. phymatum *and *B. phytofirmans*, 17 in *B. xenovorans*). Conversely, *B. mallei*, *B. pseudomallei *and *B*. *thailandiensis *strains possess a lower HAE1/HME copy number, ranging from 6 to 11 in *B. mallei *and *B*. *thailandiensis *species, respectively. Lastly, the species belonging to the BCC complex and embedded in the upper monophyletic cluster of Figure 9c, possess a number of HME/HAE1 copies ranging from 11, in *B. vietnamiensis *G4 and *B. ambifaria *AMMD, to 18 in *B. cenocepacia *representatives. In addition to these data, the phylogenetic tree constructed with all the 254 retrieved sequences of the *Burkholderia *genus (Figure 7 and Additional File 3) revealed that the *Burkholderia *species are distributed all over the tree and that the monophyly of the main *Burkholderia *clade (according to the reference phylogeny of Figure 9c) is overall respected, suggesting that the *ceoB*-like sequences did not undergo massive HGT events between different *Burkholderia *species or, if this occurred, it happened in the ancestor of *Burkholderia*. However, the possibility that some of these genes might have been exchanged between strains belonging to the same or different *Burkholderia *species and/or between different DNA molecules within the same cytoplasm cannot be *a priori *excluded. Indeed, it is known that bacteria belonging to this genus harbour two-three different chromosomes and some of them are among the largest genome-sized and most versatile bacteria known. Besides, these genomes harbour a relevant number of genes coding for transposases and integrases, (the percentage content of transposases *per *genome and integrases *per *genome ranged from 2.09 in *B. lata *sp.383 to 4.06 in *B. thailandiensis *E264 and from 0.08 in *B. ambifaria *MC40-6 to 0.31 in *B. multivorans *ATCC 17616, respectively) suggesting that they might frequently undergo DNA rearrangements that, in turn, might alter their gene structure and/or organization [[61] and references therein]. In addition to this, some of the 21 *Burkholderia *strains harbour one or more large plasmids, which possess genes coding for genetic mobile elements. These elements are responsible for the flow of genes between plasmids and chromosomes inhabiting the same cytoplasm. At the same time, plasmids may also permit the spreading of metabolic traits between cells of the same or different species. Indeed, it has been recognized that HGT is one of the major forces driving the evolution of genes and genomes [62,63]. The analysis of the genomic localization of the 254 *ceoB*-like genes revealed that six of them are located in four different large plasmid molecules harboured by three different strains (Table 4). Three of these genes (two from *B. multivorans *and one from *B. vietnamiensis*) fell in Cluster B (corresponding to HME proteins). In the case of *B. multivorans *one of the two plasmid encoded sequences (gi: 161506614, Table 4) has a paralog in the chromosome. This finding opens the possibility that the two copies (the chromosome one and the plasmid one) might be the result of an internal rearrangement. However, the degree of sequence identity between them is identical (96-97%) to the one shared with the *B. vietnamiensis *plasmids encoded sequence (gi: 134287672, Table 4). So, it cannot be *a priori *excluded the possibility that the two *B. multivorans *and *B. vietnamiensis *plasmid-borne genes might have been exchanged between the two strains through plasmid-mediated HGT event(s) occurring recently during evolution. A preliminary comparative analysis of the sequences of these two plasmids revealed that very likely they could have exchanged some regions between each other (Maida et al, manuscript in preparation). The other three sequences are harboured by two *B. phymatum *plasmids. Two of them (both from plasmid pBPHY01) code for proteins (Table 4) falling in the group of sequences with uncertain function (Additional File 1) and they do not have any counterpart in the host chromosomes; thus, it is possible that these two sequences might have been moved from the chromosome to pBPHY01. The third one belongs to the HAE1 family and with its closest paralog in the chromosome [share a degree of sequence identity of 94%].

**Table 4:** *Burkholderia *plasmids harbouring *ceoB*-like genes

		Plasmid		
		
Strain	Protein	Name	Dimension (bp)	RND Cluster
*B. multivorans *ATTC17616	gi:161506614	pBMUL01	167422	CDS 12
	gi:161506504			CDS 11

*B. vietnamiensis *G4	gi:134287672	pBVIE02	2656616	CDS 12

*B. phymatum *STM815	gi:186471278	pBPHY01	1904893	CDS 15
	gi:186471940			CDS 14
	gi:186474676	pBPHY02	595102	--

The whole body of data presented here suggests a likely evolutionary model accounting for the evolution of the HME and HAE1 systems in *Burkholderia *genus. According to this model, the ancestor of all the extant *Burkholderia *already possessed a high number of HME/HAE1-like gene copies. Although it is not possible to infer the exact copy number of CeoB coding genes in the genome of the *Burkholderia *ancestor, it is possible that this number might have been close to the one exhibited by the species embedded in the cluster at the root of the *Burkholderia *reference tree in Figure 9c.

The high degree of sequence similarity shared by these different copies strongly suggests that they belong to a paralogous gene family, originated from an ancestral *ceoB*-like sequence that underwent many duplication events and existing long before the appearance of the ancestor of *Burkholderia*. On the basis of the available data, it is not possible to infer whether this ancestor gene was organized in operon with an OMP and/or MFP coding gene. However, the finding that most of the *ceoB*-like genes are organized into operons and that (at least) three different operon structures exist in *B. cenocepacia *J2315 genome, might suggest the existence of three different operon organizations in the genome of the *Burkholderia *ancestor. The possible number of each operon is still unknown and their study is beyond the scope of this work. A preliminary analysis performed on other β-proteobacterial subdivision revealed a similar pattern of both RND copy number and operon structure (data not shown). If this idea is correct, then, starting from this ancestral gene pool, multiple events of gene duplication and gene loss would have led to the copy number patterns of the extant *Burkholderia *representatives. Accordingly, those species possessing the lowest number of HME/HAE1 related sequences (*B. mallei *and *B. pseudomallei *strains) are those for which massive genome reduction (and consequently gene loss) has been documented [64]. Regarding the function of ancestral HME/HAE1-like proteins, it is not possible, standing to data presented in this work, to infer whether they were already specialized in recognizing a specific substrate or not. However, it can be mentioned the hypothesis that these ancestral efflux pumps might have been able to recognize different substrates, hence exhibiting low substrate specificity. This is in agreement with the notion that some of the efflux pumps are able to interact with different substrates. This latter observation fits quite well with a recently proposed idea [10] according to which MDR proteins (hence including HME/HAE1-like systems) might be involved in extruding structurally related (non metabolised) substrates out of the cell, thus functioning as safety valves. Duplication events, followed by evolutionary divergence might have concurred in refining their substrate specificity, allowing them to selectively extrude out of the cell a given chemical compound (antibiotics or heavy-metals). This idea represents a further validation (and an extension) of the "patchwork" hypothesis, originally proposed by Jensen [65], to explain the origin and evolution of enzymes involved in metabolic pathways [18,66].

## Conclusion

In this work we have performed a comprehensive comparative analysis of the HME and HAE1 efflux systems in *Burkholderia *genus. A total of 254 coding sequences were retrieved from the available *Burkholderia *sequenced genomes and analyzed at different levels, adopting different bioinformatic tools. A deep phylogenetic analysis, in which experimentally characterized sequences were also included, permitted to assign a putative function (i.e. antibiotic resistance, heavy metal efflux) to (up to now) uncharacterized *Burkholderia *sequences. Furthermore, the analysis of conserved residues involved in different functions (substrate recognition, proton translocation) of HME and HAE1 sequences allowed refining motifs previously identified on the basis of a smaller protein dataset. Given the high variability in the number of HAE1 and HME coding sequences found in extant *Burkholderia *species, we tried to correlate both the number and the types (i.e. the transported substrate) with the different characteristics observed in the *Burkholderia *strains (pathogenic lifestyle, genome size, colonized habitat). However, no apparent correlation emerged, suggesting that other forces might be responsible in determining the types and the copy number of HME/HAE1 sequences in the *Burkholderia *genus. Remarkably, we observed that only HAE1 proteins are mainly responsible for the different number of proteins observed in strains of the same species. By assuming that the physiological role of these proteins is resistance to one or more antibiotics, this finding, in turn, may suggest that the acquisition of antibiotic resistance might be the main selective pressure driving the expansion of this protein family. On the other hand, these proteins might play other roles relevant to the behaviour of bacteria in their natural ecosystems, so other selective pressure might drive the evolution of this protein family. Data concerning both the distribution and the phylogenetic analysis of the HAE1 and HME in the genus *Burkholderia *allowed depicting a likely evolutionary model accounting for the evolution and spreading of HME and HAE1 systems in *Burkholderia *genus. The occurrence of several species-specific duplication and gene and/or operon loss events finally led to the extant pattern of copy number/type observed in modern-day *Burkholderia*.

It would be interesting to individuate specific residues directly involved in substrate binding. Some data concerning this issue have been obtained studying *E. coli *AcrB protein [26,67-71]. However, these experiments revealed the existence of a set of residues possibly involved in substrate binding, but none of them appeared to be *per se *essential for substrate binding [26,67-71]. A preliminary analysis of the overall *Burkholderia *sequences dataset did not reveal a strong conservation of the same key residues found in the *E. coli *AcrB sequences (data not shown). At least two different explanations can be proposed for this scenario: i) the first refers to the fact that the differences observed within *E. coli *AcrB and *Burkholderia *AcrB-like sequences might be due to the phylogenetic distance existing between them, thus not reflecting differences in the mechanism of substrate binding/recognition of the corresponding transporters. The availability of similar experimental data in a *Burkholderia *cellular background will provide important insights about this issue; ii) the second one takes into account the possibility that the correct substrate binding might rely on a set of (interchangeable) residues rather than on single specific position.

Lastly, the whole data presented in this work may serve as a basis for future experimental tests, focused especially on HAE1 proteins, aimed at the identification of novel targets in antimicrobial therapy against *Burkholderia *species.

## Methods

### Sequence retrieval

Amino acid sequences from the 21 completely sequenced genomes of strains belonging to the genus *Burkholderia*, available on 1^st ^May 2009, were retrieved from GenBank database http://www.ncbi.nlm.nih.gov (Table 3). BLAST [72] probing of database was performed with the BLASTP option of this program using default parameters. Only those sequences retrieved at an E-value below the 0.05 threshold were taken into account. 16S rRNA gene nucleotide sequences were retrieved from Ribosomal Database Project http://rdp.cme.msu.edu/[73].

### Sequence alignment

The ClustalW [47] program in the BioEdit [74] package and the Muscle program [75] were used to perform pairwise and multiple amino acid sequence alignments. Alignments were manually checked and mis-aligned regions were removed.

### Phylogenetic analysis

Neighbor-Joining (NJ) phylogenetic trees were obtained with Mega 4 software [76], complete deletion option and 1000 bootstraps replicates. Maximum Likelihood phylogenetics trees were constructed using Phyml [77], with a WAG model of amino acid substitution, including a gamma function with 6 categories to take into account differences in evolutionary rates at sites. Statistical support at nodes was obtained by non-parametric bootstrapping on 1000 re-sampled datasets by using Phyml.

### Hydropathy plot

Hydropathy plots were obtained on Protscale website http://www.expasy.ch/tools/protscale.html[46] using Kyte and Doolittle scale [45].

### Residues conservation

Analyisis of conservation of amino acid residues was performed using the Weblogo application http://weblogo.berkeley.edu/ using default parameters [78].

## Abbreviations

APPE: Aryl Polyene Pigment Exporter; BCC: *Burkholderia cepacia *complex; CF: Cystic fibrosis; E: Enviromental; EST: Eukaryotic (putative) Sterol transporter; H: Host; HAE-1: Hydrophobe Amphiphile Efflux-1; HAE-2: Hydrophobe-Amphiphile Efflux-2; HAE-3: Hydrophobe-Amphiphile Efflux-3; HGT: Horizontal Gene Transfer; HME: Heavy-Metal Efflux; Mbp: Mega Base Pair; MFP: Membrane Fusion Protein; NC: Not classified; NFE: putative Nodulation Factor Exporter; NJ: Neighbor-Joining; OMP: Outer-Membrane Protein; P: Pathogen; RND: Resistance-Nodulation-Cell Division; SecDF: Secretion System DF; TCDB: Transport Classification Database; TMS: TransMembrane Spanner.

## Authors' contributions

EP, MF, RF conceived the study. EP and MF performed the analyses. All authors discussed data and drafted the manuscript. All authors read and approved the final manuscript.

## Supplementary Material

Additional File 1 **Phylogenetic tree**. Phylogenetic tree constructed using the 254 *Burkholderia *CeoB-like sequences Click here for file

Additional File 2 **Table of characterized RND proteins**. Table of 62 characterized RND proteins and their relative substrate Click here for file

Additional File 3 **Phylogenetic tree**. Phylogenetic tree constructed using the 254 *Burkholderia *CeoB-like sequences plus sequences of characterized proteins. Click here for file

Additional File 4 **Relationship between RND proteins and lifestyle and pathogenicity**. Relationship between RND proteins and lifestyle and pathogenicity Click here for file
