# Supplementary material for: Exploring the HME and HAE1 efflux systems in the genus Burkholderia
Source: BMC Evol Biol. 2010 Jun 3;10:164. doi: 10.1186/1471-2148-10-164 (PMC2891726; doi:10.1186/1471-2148-10-164)
Supplement: Additional File 1 — Phylogenetic tree. Phylogenetic tree constructed using the 254 Burkholderia CeoB-like sequences [file 1471-2148-10-164-S1.PPT]

## Slide 1
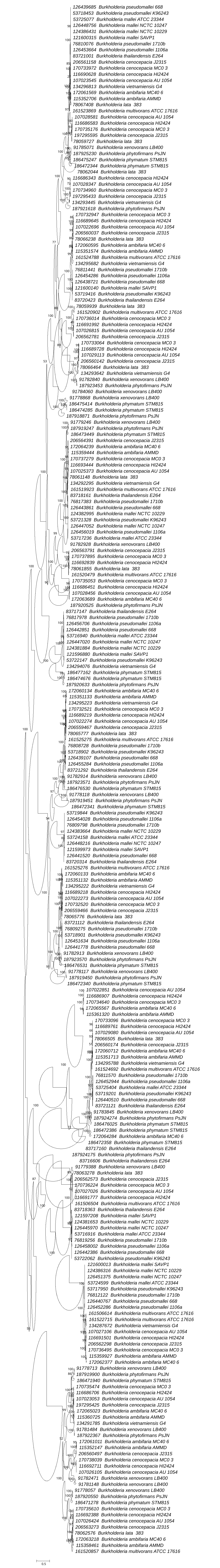

126439685 Burkholderia pseudomallei 668
 53718453 Burkholderia pseudomallei K96243
 53725077 Burkholderia mallei ATCC 23344
 126448756 Burkholderia mallei NCTC 10247
 124386431 Burkholderia mallei NCTC 10229
 121600315 Burkholderia mallei SAVP1
 76810076 Burkholderia pseudomallei 1710b
 126453664 Burkholderia pseudomallei 1106a
 83721001 Burkholderia thailandensis E264
 206561158 Burkholderia cenocepacia J2315
 170733972 Burkholderia cenocepacia MC0 3
 116690628 Burkholderia cenocepacia HI2424
 107023545 Burkholderia cenocepacia AU 1054
 134296813 Burkholderia vietnamiensis G4
 172061569 Burkholderia ambifaria MC40 6
 115352706 Burkholderia ambifaria AMMD
 78067408 Burkholderia lata 383
 161523869 Burkholderia multivorans ATCC 17616
 107028581 Burkholderia cenocepacia AU 1054
 116686583 Burkholderia cenocepacia HI2424
 170735176 Burkholderia cenocepacia MC0 3
 197295595 Burkholderia cenocepacia J2315
 78059727 Burkholderia lata 383
 91785071 Burkholderia xenovorans LB400
 187925230 Burkholderia phytofirmans PsJN
 186475247 Burkholderia phymatum STM815
 186472344 Burkholderia phymatum STM815
 78062044 Burkholderia lata 383
 116686343 Burkholderia cenocepacia HI2424
 107028347 Burkholderia cenocepacia AU 1054
 170734960 Burkholderia cenocepacia MC0 3
 197295433 Burkholderia cenocepacia J2315
 134293445 Burkholderia vietnamiensis G4
 187921618 Burkholderia phytofirmans PsJN
 170732947 Burkholderia cenocepacia MC0 3
 116689645 Burkholderia cenocepacia HI2424
 107022696 Burkholderia cenocepacia AU 1054
 206560037 Burkholderia cenocepacia J2315
 78066238 Burkholderia lata 383
 172060595 Burkholderia ambifaria MC40 6
 115351574 Burkholderia ambifaria AMMD
 161524788 Burkholderia multivorans ATCC 17616
 134295682 Burkholderia vietnamiensis G4
 76811441 Burkholderia pseudomallei 1710b
 126454286 Burkholderia pseudomallei 1106a
 126438721 Burkholderia pseudomallei 668
 121600140 Burkholderia mallei SAVP1
 53719416 Burkholderia pseudomallei K96243
 83720423 Burkholderia thailandensis E264
 78059939 Burkholderia lata 383
 161520902 Burkholderia multivorans ATCC 17616
 170736014 Burkholderia cenocepacia MC0 3
 116691992 Burkholderia cenocepacia HI2424
 107026815 Burkholderia cenocepacia AU 1054
 206562781 Burkholderia cenocepacia J2315
 170733064 Burkholderia cenocepacia MC0 3
 116689728 Burkholderia cenocepacia HI2424
 107029113 Burkholderia cenocepacia AU 1054
 206560142 Burkholderia cenocepacia J2315
 78066464 Burkholderia lata 383
 134293642 Burkholderia vietnamiensis G4
 91782840 Burkholderia xenovorans LB400
 187923453 Burkholderia phytofirmans PsJN
 91784060 Burkholderia xenovorans LB400
 91778868 Burkholderia xenovorans LB400
 186475414 Burkholderia phymatum STM815
 186474285 Burkholderia phymatum STM815
 187918871 Burkholderia phytofirmans PsJN
 91779246 Burkholderia xenovorans LB400
 187919247 Burkholderia phytofirmans PsJN
98
100
85
100
27
100
100
100
56
97
100
97
100
100
100
100
98
95
100
90
72
100
100
100
88
100
93
99
86
77
97
51
99
100
100
100
100
80
100
100
49
100
89
99
100
100
97
53
100
100
100
100
100
100
100
100
91
66
100
97
91
100
100
100
100
100
99
72
100
39
100
 126452286 Burkholderia pseudomallei 1106a
 161506614 Burkholderia multivorans ATCC 17616
 161522715 Burkholderia multivorans ATCC 17616
 134287672 Burkholderia vietnamiensis G4
 107027106 Burkholderia cenocepacia AU 1054
 116691501 Burkholderia cenocepacia HI2424
 206562298 Burkholderia cenocepacia J2315
 170736495 Burkholderia cenocepacia MC0 3
 115359927 Burkholderia ambifaria AMMD
 172062377 Burkholderia ambifaria MC40 6
 91778713 Burkholderia xenovorans LB400
 187919900 Burkholderia phytofirmans PsJN
 186471940 Burkholderia phymatum STM815
 170735474 Burkholderia cenocepacia MC0 3
 116686706 Burkholderia cenocepacia HI2424
 107023053 Burkholderia cenocepacia AU 1054
 197295425 Burkholderia cenocepacia J2315
 172065023 Burkholderia ambifaria MC40 6
 115360725 Burkholderia ambifaria AMMD
 134291785 Burkholderia vietnamiensis G4
 91781484 Burkholderia xenovorans LB400
 187922367 Burkholderia phytofirmans PsJN
 172061011 Burkholderia ambifaria MC40 6
 115352147 Burkholderia ambifaria AMMD
 206560497 Burkholderia cenocepacia J2315
 170738039 Burkholderia cenocepacia MC0 3
 116692711 Burkholderia cenocepacia HI2424
 107026105 Burkholderia cenocepacia AU 1054
 91782471 Burkholderia xenovorans LB400
 91781148 Burkholderia xenovorans LB400
 91778057 Burkholderia xenovorans LB400
 187920550 Burkholderia phytofirmans PsJN
 186471278 Burkholderia phymatum STM815
 170735610 Burkholderia cenocepacia MC0 3
 116692388 Burkholderia cenocepacia HI2424
 107026424 Burkholderia cenocepacia AU 1054
 206563273 Burkholderia cenocepacia J2315
 78062576 Burkholderia lata 383
 172063218 Burkholderia ambifaria MC40 6
 115358461 Burkholderia ambifaria AMMD
 161520857 Burkholderia multivorans ATCC 17616
 186473449 Burkholderia phymatum STM815
 206564391 Burkholderia cenocepacia J2315
 172064239 Burkholderia ambifaria MC40 6
 115359444 Burkholderia ambifaria AMMD
 170737279 Burkholderia cenocepacia MC0 3
 116693444 Burkholderia cenocepacia HI2424
 107025373 Burkholderia cenocepacia AU 1054
 78061148 Burkholderia lata 383
 134292295 Burkholderia vietnamiensis G4
 161519923 Burkholderia multivorans ATCC 17616
 83718161 Burkholderia thailandensis E264
 76817383 Burkholderia pseudomallei 1710b
 126443861 Burkholderia pseudomallei 668
 124382995 Burkholderia mallei NCTC 10229
 53721328 Burkholderia pseudomallei K96243
 126447052 Burkholderia mallei NCTC 10247
 126456019 Burkholderia pseudomallei 1106a
 53717236 Burkholderia mallei ATCC 23344
 91782928 Burkholderia xenovorans LB400
 206563791 Burkholderia cenocepacia J2315
 170737895 Burkholderia cenocepacia MC0 3
 116692839 Burkholderia cenocepacia HI2424
 78061855 Burkholderia lata 383
 161520479 Burkholderia multivorans ATCC 17616
 170735053 Burkholderia cenocepacia MC0 3
 116686451 Burkholderia cenocepacia HI2424
 107028456 Burkholderia cenocepacia AU 1054
 172063689 Burkholderia ambifaria MC40 6
 187920525 Burkholderia phytofirmans PsJN
 83717147 Burkholderia thailandensis E264
 76817978 Burkholderia pseudomallei 1710b
 126456706 Burkholderia pseudomallei 1106a
 126442851 Burkholderia pseudomallei 668
 53716940 Burkholderia mallei ATCC 23344
 126447020 Burkholderia mallei NCTC 10247
 124381884 Burkholderia mallei NCTC 10229
 121596880 Burkholderia mallei SAVP1
 53722147 Burkholderia pseudomallei K96243
 134294076 Burkholderia vietnamiensis G4
 186477162 Burkholderia phymatum STM815
 186474676 Burkholderia phymatum STM815
 187920633 Burkholderia phytofirmans PsJN
 172060134 Burkholderia ambifaria MC40 6
 115351133 Burkholderia ambifaria AMMD
 134295223 Burkholderia vietnamiensis G4
 170732521 Burkholderia cenocepacia MC0 3
 116689219 Burkholderia cenocepacia HI2424
 107022274 Burkholderia cenocepacia AU 1054
 206559467 Burkholderia cenocepacia J2315
 78065777 Burkholderia lata 383
 161525275 Burkholderia multivorans ATCC 17616
 76808728 Burkholderia pseudomallei 1710b
 53718902 Burkholderia pseudomallei K96243
 126439107 Burkholderia pseudomallei 668
 126455284 Burkholderia pseudomallei 1106a
 83721292 Burkholderia thailandensis E264
 91782914 Burkholderia xenovorans LB400
 187923571 Burkholderia phytofirmans PsJN
 186476530 Burkholderia phymatum STM815
 91778118 Burkholderia xenovorans LB400
 187919451 Burkholderia phytofirmans PsJN
 186472341 Burkholderia phymatum STM815
 53719844 Burkholderia pseudomallei K96243
 126454028 Burkholderia pseudomallei 1106a
 76809798 Burkholderia pseudomallei 1710b
 124383664 Burkholderia mallei NCTC 10229
 53724158 Burkholderia mallei ATCC 23344
 126448216 Burkholderia mallei NCTC 10247
 121599973 Burkholderia mallei SAVP1
 126441520 Burkholderia pseudomallei 668
 83720314 Burkholderia thailandensis E264
100
100
100
90
96
100
100
100
96
100
100
100
99
100
100
65
68
95
100
97
100
90
100
 161525276 Burkholderia multivorans ATCC 17616
 172060133 Burkholderia ambifaria MC40 6
 115351132 Burkholderia ambifaria AMMD
 134295222 Burkholderia vietnamiensis G4
 116689218 Burkholderia cenocepacia HI2424
 107022273 Burkholderia cenocepacia AU 1054
 170732520 Burkholderia cenocepacia MC0 3
 206559466 Burkholderia cenocepacia J2315
 78065776 Burkholderia lata 383
 83721112 Burkholderia thailandensis E264
 76809275 Burkholderia pseudomallei 1710b
 53718901 Burkholderia pseudomallei K96243
 126451634 Burkholderia pseudomallei 1106a
 126441778 Burkholderia pseudomallei 668
 91782913 Burkholderia xenovorans LB400
 187923570 Burkholderia phytofirmans PsJN
 186476531 Burkholderia phymatum STM815
 91778117 Burkholderia xenovorans LB400
 187919450 Burkholderia phytofirmans PsJN
 186472340 Burkholderia phymatum STM815
 107022851 Burkholderia cenocepacia AU 1054
 116686907 Burkholderia cenocepacia HI2424
 170734640 Burkholderia cenocepacia MC0 3
 172065567 Burkholderia ambifaria MC40 6
 115361320 Burkholderia ambifaria AMMD
 170733096 Burkholderia cenocepacia MC0 3
 116689761 Burkholderia cenocepacia HI2424
 107029080 Burkholderia cenocepacia AU 1054
 78066505 Burkholderia lata 383
 206560174 Burkholderia cenocepacia J2315
 172060712 Burkholderia ambifaria MC40 6
 115351713 Burkholderia ambifaria AMMD
 134295788 Burkholderia vietnamiensis G4
 161524692 Burkholderia multivorans ATCC 17616
 76811570 Burkholderia pseudomallei 1710b
 126452944 Burkholderia pseudomallei 1106a
 53725404 Burkholderia mallei ATCC 23344
 53719201 Burkholderia pseudomallei K96243
 126440510 Burkholderia pseudomallei 668
 83721121 Burkholderia thailandensis E264
 91783845 Burkholderia xenovorans LB400
 187924274 Burkholderia phytofirmans PsJN
 186476025 Burkholderia phymatum STM815
 186472386 Burkholderia phymatum STM815
 172064284 Burkholderia ambifaria MC40 6
 186472358 Burkholderia phymatum STM815
 83717160 Burkholderia thailandensis E264
 187924175 Burkholderia phytofirmans PsJN
 83716606 Burkholderia thailandensis E264
 91779388 Burkholderia xenovorans LB400
 78063278 Burkholderia lata 383
 206562573 Burkholderia cenocepacia J2315
 170736224 Burkholderia cenocepacia MC0 3
 107027026 Burkholderia cenocepacia AU 1054
 116691777 Burkholderia cenocepacia HI2424
 161506504 Burkholderia multivorans ATCC 17616
 83718363 Burkholderia thailandensis E264
 121597208 Burkholderia mallei SAVP1
 124381653 Burkholderia mallei NCTC 10229
 126445970 Burkholderia mallei NCTC 10247
 53716916 Burkholderia mallei ATCC 23344
 76819256 Burkholderia pseudomallei 1710b
 126458002 Burkholderia pseudomallei 1106a
 126442386 Burkholderia pseudomallei 668
 53722062 Burkholderia pseudomallei K96243
 121600013 Burkholderia mallei SAVP1
 124386316 Burkholderia mallei NCTC 10229
 126451375 Burkholderia mallei NCTC 10247
 53724599 Burkholderia mallei ATCC 23344
 53717950 Burkholderia pseudomallei K96243
 76812122 Burkholderia pseudomallei 1710b
 126440767 Burkholderia pseudomallei 668
100
100
100
100
100
100
100
97
100
54
97
83
100
100
59
100
100
100
26
100
93
100
95
100
100
100
100
100
100
91
100
100
100
100
94
96
66
93
51
100
100
100
100
100
100
100
100
100
100
100
100
72
100
89
87
100
92
100
100
84
92
97
100
100
100
100
97
55
93
95
99
100
100
100
86
45
100
100
100
100
99
100
72
100
52
95
100
100
100
100
100
100
100
100
100
95
83
100
97
100
100
100
0.5
