# Supplementary material for: Exploring the HME and HAE1 efflux systems in the genus Burkholderia
Source: BMC Evol Biol. 2010 Jun 3;10:164. doi: 10.1186/1471-2148-10-164 (PMC2891726; doi:10.1186/1471-2148-10-164)
Supplement: Additional File 2 — Table of characterized RND proteins. Table of 62 characterized RND proteins and their relative substrate [file 1471-2148-10-164-S2.DOC]

| **ORGANISM** | **STRAIN** | **PROTEIN NAME** | **FAMILY** | **SUBSTRATE** |
| --- | --- | --- | --- | --- |
| *Acinetobacter baumannii* | BM4454 | AdeB | HAE_1 | Aminoglycosides, Chloramphenicol, Fluoroquinolones, Novobiocin, Tetracycline, Trimethoprim, Erythromycin, some β-lactams, EtBr e Tigecycline [1] |
| *Acinetobacter baumannii* | BM4454 | AdeJ | HAE_1 | β-lactams, Chloramphenicol, Tetracycline, Erythromycin, Lincosamides, Fluoroquinolones, Fusidic Acid, Novobiocin, Rifampin, Trimethoprim, Acridine, Safranin, Pyronine, SDS [2] |
| *Agrobacterium tumefaciens* | 1D1609 | IfeB | HAE_1 | Isoflavenoid [TCDB] [3] |
| *Brucella suis* |  | BepE | HAE_1 | Deoxycholate (DOC), EtBr, Crystal violet, Ampicillin, Norfloxacin, Ciprofloxacin, Novobiocin, Polymixin B, Tetrayicline, Doxicycline, Thiamphenicol, Acriflavin, SDS [4] |
| *Brucella suis* |  | BepG | HAE_1 | DOC, SDS, Nalidixic Acid, in the absence of BepE [4] |
| *Burkholderia cenocepacia* | J2315 | CeoB | HAE_1 | Chloramphenicol, Trimethoprim, Ciprofloxacin [5] [6] |
| *Burkholderia cenocepacia* | J2315 | Orf2 | HAE_1 | Fluoroquinolones, Tetraphenylphosphonium, Streptomycin, EtBr [7] |
| *Burkholderia glumae* | BGR1 | ToxH | HAE_1 | Toxoflavin [TCDB] [8] |
| *Burkholderia pseudomallei* | 1026b | AmrB | HAE_1 | Aminoglicosides, Macrolides [TCDB] [9] |
| *Burkholderia pseudomallei* | ATCC23343 | BpeB | HAE_1 | β-lactams, Aminoglicosides, Macrolides, Acriflavin [10] [11] it also export various compounds implicated in *Quorum sensing* [12] |
| *Burkholderia pseudomallei* | K96293 | BpeF | HAE_1 | Chloramphenicol, Trimethoprim [13] |
| *Campylobacter jejuni* | 81-176 | CmeB | HAE_1 | β-lactams, Fluoroquinolones, Macrolides, Chloramphenicol, Tetracycline, EtBr, Acridine orange , SDS, Cefotaxime, Rifampicin, Erythromycin, Salicylate [TCDB] [14] |
| *Enterobacter aerogens* | BW16627 | EefB | HAE_1 | Chloramphenicol, Ciprofloxacin, Erythromycin, Tetracycline, Doxycycline [TCDB] [15] |
| *Enterobacter cloacae* |  | AcrB | HAE_1 | Tigecycline [16] |
| *Escherichia coli* | K12 | AcrB | HAE_1 | β-lactams, Chloramphenicol, Fluoroquinolones, Macrolides, Novobiocin, Rifampicin [1] Tetracycline, Erythromycin, Nalidixic Acid, Fusidic Acid, Doxorubicin, Trimethoprim, Acriflavin, Crystal violet, EtBr, Rhodamine 6-G, TPP, Benzalkonium, SDS, Triton X-100, Deoxicholate, Bile salt, Organic solvents (Alkanes), Growth inhibitory steroid hormones, Phospholipids [TCDB] |
| *Escherichia coli* | K12 | AcrD | HAE_1 | Aminoglicosides, Fusidic Acid [1] [17] [18]SDS, Deoxicholate, Growth inhibitory steroid hormones [TCDB] |
| *Escherichia coli* | K12 | AcrF | HAE_1 | Fluoroquinolones [1] Acriflavin, Doxorubicin, EtBr, Rhodamine 6G, SDS, Deoxicholate [TCDB] |
| *Escherichia coli* | K12 | MdtB | HAE_1 | Novobiocin [1] [19] [20] Nalidixic Acid, Norfloxacin, Enoxacin, Kanamycin, Benzalkonium, SDS, deoxicholate (Also contribuites to copper and zinc resistance; regulation is mediated by BaeSR , and indole, Copper and Silver induce) [TCDB] |
| *Escherichia coli* | K12 | MdtC | HAE_1 | Novobiocin [1] [19] [20] Bile salt [TCDB] [19] |
| *Escherichia coli* | K12 | MdtF (YhiV) | HAE_1 | Novobiocin [1] [21] Erythromycin, Doxorubicin, Crystal violet, EtBr, Rhodamine 6G, TPP, Benzalkonium, SDS, Deoxicholate, growth inhbitory steroid hormones [TCDB] |
| *Escherichia coli* | K12 | CusA | HAE_1 | Copper and Silver [22] [TCDB] |
| *Francisella tularensis* | Schu S4 | AcrB | HAE_1 | Ampicillin, Carbenicillin, Cefoperazone, Rifampicin, Tetracycline, SDS, Triton X-100, Deoxycholate, Cholate [TCDB] [23] |
| *Haemophilus influenzae* | Rd KW20 | AcrB | HAE_1 | Erythromycin, Rifampin, Novobiocin, EtBr, Crystal violet [24] |
| *Klebsiella pneumonie* |  | AcrB | HAE_1 | Floroquinolones [1] |
| *Neisseria gonorrhoeae* |  | MtrD | HAE_1 | Fatty acid, Bile salts, Gonadal steroid, Antibacterial peptide [TCDB] [25] No Ciprofloxacin e Streptomycin [26] |
| *Porphyromonas gingivalis* | ATCC 33277 | XepB | HAE_1 | EtBr, Puromycin, Rifampin, Norfloxacin, Ofloxacin, Ciprofloxacin, Tetracycline, Minocycline, Berberine, Acriflavine, SDS [27] |
| *Proteus mirabilis* |  | AcrB | HAE_1 | Novobiocin [1] [28] |
| *Pseudomonas aeruginosa* | PA01 | MexB | HAE_1 | Aminoglycosides, β-lactams, Chloramphenicol, Macrolides, Novobiocin, Tetracycline, Trimethoprim [1] [29] Fluoroquinolones, Biocides, CBR-4830 [TCDB] β-lactams inhibitors, Triclorosan, EtBr, SDS, Aromatic hydrocarbons, Thiolactomycin, Cerulein, Acyleted homoserine lactones [30] |
| *Pseudomonas aeruginosa* | PA01 | MexD | HAE_1 | Chloramphenicol, Cefalosporin, Fluoroquinolones, Tetracycline [1] [31] β-lactams, Macrolides, Biocides [TCDB] [32] Novobiocin, Trimethoprim, Crystal violet, EtBr, Acriflavin, SDS, Aromatic hydrocarbons, Triclorosan [30] |
| *Pseudomonas aeruginosa* | PA01 | MexF | HAE_1 | Chloramphenicol, Fluoroquinolones [1] [33] Biocides, Xenobiotics [TCDB] [33] [34] Trimethoprim, Aromatic hydrocarbons, Triclosan, Pseudomonas quinolone signal [TCDB] [33] |
| *Pseudomonas aeruginosa* | PA01 | MexI | HAE_1 | Novobiocin [1] [35] [36] Fluoroquinolones [TCDB] [34] Vanadium, Acylated homoserine lactones [30] |
| *Pseudomonas aeruginosa* | PA01 | MexN | HAE_1 | Chloramphenicol, Triamphenicol [37] [TCDB] [34] |
| *Pseudomonas aeruginosa* | PA01 | MexK | HAE_1 | Erytromycin, Tetracyclin [1] [38] Fluoroquinolones, Tetracycline, Macolides, Chloramphenicol, Biocides, Triclosan, [TCDB] [34] [34] [30] |
| *Pseudomonas aeruginosa* | PA01 | MexQ | HAE_1 | Macrolides, Fluoroquinolones [37] Tetracycline, Chloramphenicol [TCDB] [34] |
| *Pseudomonas aeruginosa* | PA01 | MexW | HAE_1 | Chloramphenicol, Fluoroquinolones, Tetracycline [1] [39] Macrolides [TCDB] [34] |
| *Pseudomonas aeruginosa* | PA01 | MexY | HAE_1 | Aminoglycosides, Macrolides, Tetracycline [1] [40] β-lactams, Fluoroquinolones, Chloramphenicol, Erythromycin, Ofloxacin [TCDB] [41] [30] |
| *Pseudomonas aeruginosa* | PA01 | CzrA | HME | Cadmium, Zinc [30] [42] [43] |
| *Pseudomonas aeruginosa* | PA01 | TriC | HAE_1 | Triclosan [TCDB] [44] |
| *Pseudomonas fluorescence* |  | EmhB | HAE_1 | Polycyclic aromatic hydrocarbon, Chloramphenicol, Nalidixic acid [TCDB] [45] |
| *Pseudomonas fluorescence* |  | CztA | HME | Cadmium, Zinc [46] |
| *Pseudomonas putida* | DOT-T1E | TtgB | HAE_1 | Toluene, Chloramphenicol, Tetracycline, Nalidixic acid, Norfloxacin, Streptomycin, Ampicillin, Cefotaxime, Plant secondary products with antimicrobial properties, Biocides, EtBr [47] [48] [49]Stirene, m-xylene, ethylbenzene, propylbenzene [TCDB] |
| *Pseudomonas putida* | DOT-T1E | TtgE | HAE_1 | Toluene, Styrene [50] [TCDB] |
| *Pseudomona putida* | DOT-T1E | TtgH | HAE_1 | Toluene, Styrene, m-xylene, ethyllbenzene, propylbenzene [50] [TCDB] |
| *Pseudomona putida* | KT2440 | CzcA1 | HME | Zinc, Cadmium, Lead [TCDB] [51] |
| *Pseudomona putida* | KT2440 | CzcA2 | HME | Zinc [51] |
| *Pseudomona putida* | S12 | SrpB | HAE_1 | Organic solvents (Toluene) [TCDB] [52] |
| *Pseudomona putida* | S12 | ArpB | HAE_1 | Tetracycline, Chloramphenicol, Carbenicillin, Streptomicyn, Erythromycin, Novobiocin, etc. [TCDB] [53] |
| *Pseudomonas syringae pv. Phaseolicola* | 1448A | MexB | HAE_1 | Acridine orange, Acriflavin, Ampicillin, Benzalkonium chloride, Berberine, Carbenicillin, Cefoperazone, Chloramphenicol, Ciprofloxacin, Clindamicyn, Crystal violet, Daunorubicin, Erythromycin, EtBr, Fusaric acid, Fusidic acid, Kanamycin, Mitomycin C, Nalidixic acid, Naringenin, Nitrofurantoin, Norfloxacin, Novobiocin, Phloretin, Piperacillin, Puromicyn, Rodamine 6G, Tetracycline, Tetraphenylphosphonium chloride, Trimethoprim [54] |
| *Pseudomonas syringae pv. Syringae* | B728a | MexB | HAE_1 | Acridine orange, Acriflavin, Ampicillin, Benzalkonium chloride, Berberine, Carbenicillin, Cefoperazone, Chloramphenicol, Ciprofloxacin, Clindamicyn, Crystal violet, Daunorubicin, Erythromycin, EtBr, Fusaric acid, Fusidic acid, Kanamycin, Mitomycin C, Nalidixic acid, Naringenin, Nitrofurantoin, Norfloxacin, Novobiocin, Phloretin, Piperacillin, Puromicyn, Rodamine 6G, Tetracycline, Tetraphenylphosphonium chloride, Trimethoprim [54] |
| *Pseudomonas syringae pv. Tomato* | DC3000 | MexB | HAE_1 | Acridine orange, Acriflavin, Ampicillin, Benzalkonium chloride, Berberine, Carbenicillin, Cefoperazone, Chloramphenicol, Ciprofloxacin, Clindamicyn, Crystal violet, Daunorubicin, Erythromycin, EtBr, Fusaric acid, Fusidic acid, Kanamycin, Mitomycin C, Nalidixic acid, Naringenin, Nitrofurantoin, Norfloxacin, Novobiocin, Phloretin, Piperacillin, Puromicyn, Rodamine 6G, Tetracycline, Tetraphenylphosphonium chloride, Trimethoprim [54] |
| *Ralstonia eutropha* |  | CzcA | HME | Cobalt, Zinc, Cadmium [TCDB] |
| *Ralstonia metallidurans* | CH34 | CnrA | HME | Nickel, Cobalt [55] [TCDB] |
| *Ralstonia metallidurans* | CH34 | NccA | HME | Cobalt, Zinc, Cadmium [56] |
| *Salmonella enterica* |  | GesB | HAE_1 | Gold [TCDB] [57] |
| *Salmonella typhimurium* |  | SilA | HME | Silver [TCDB] |
| *Serratia marcescens* |  | SdeB | HAE_1 | Chloramphenicol, Fluoroquinolones [1] SDS, EtBr, n-hexane [58] |
| *Serratia marcescens* |  | SdeY | HAE_1 | Fluoroquinolones, Tetracycline [1] Erythromycin, Benzalkonium chloride, EtBr Acriflavine, Rhodamine 6 G [59] |
| *Stenotrophomonas maltophilia* |  | SmeB | HAE_1 | Aminoglicosides, β-lactams, Fluoroquinoles [1] [60] |
| *Stenotrophomonas maltophilia* |  | SmeE | HAE_1 | Erythromycin, Fluoroquinolones, Tetrayicline [1] [61] [62] |
| *Stenotrophomonas maltophilia* | K279a | SmeJ-K | HAE_1 | Gentamicin, Amikacin, Aztreonam, Tetrayicline, Minocycline, Ciprofloxacin [63] |
| *Stenotrophomonas maltophilia* | K279a | SmeZ | HAE_1 | Gentamicin, Kanamycina, Amikacin, Tobramycin, Aztreonam [63] |
| *Vibrio cholerae* | N16961 | VexB | HAE_1 | Bile salts, SDS, Triton X-100, Polymyxin B, Erytromycine, Penicillin [64] |
| *Vibrio cholerae* | N16961 | VexD | HAE_1 | Bile salts [64] |
| *Vibrio cholerae* | N16961 | VexK | HAE_1 | SDS, Triton X-100, Bile salts [64] |
| *Vibrio cholerae* | NCTC4716 | VexF | HAE_1 | Various antimicrobials; EtBr efflux is sodium-dependent [TCDB] |
| *Vibrio parahaemolyticus* |  | VmeB | HAE_1 | Hoechst 33342, Oxacillin, Acriflavine, EtBr [TCDB] [65] |

1. Wieczorek P, Sacha P, Hauschild T, Zorawski M, Krawczyk M, Tryniszewska E: **Multidrug resistant *Acinetobacter baumannii*--the role of AdeABC (RND family) efflux pump in resistance to antibiotics**. *Folia Histochem Cytobiol* 2008, **46**(3):257-267.

2. Damier-Piolle L, Magnet S, Bremont S, Lambert T, Courvalin P: **AdeIJK, a resistance-nodulation-cell division pump effluxing multiple antibiotics in *Acinetobacter baumannii***. *Antimicrob Agents Chemother* 2008, **52**(2):557-562.

3. Palumbo JD, Kado CI, Phillips DA: **An isoflavonoid-inducible efflux pump in *Agrobacterium tumefaciens* is involved in competitive colonization of roots**. *J Bacteriol* 1998, **180**(12):3107-3113.

4. Martin FA, Posadas DM, Carrica MC, Cravero SL, O'Callaghan D, Zorreguieta A: **Interplay between two RND systems mediating antimicrobial resistance in *Brucella suis***. *J Bacteriol* 2009, **191**(8):2530-2540.

5. Burns JL, Wadsworth CD, Barry JJ, Goodall CP: **Nucleotide sequence analysis of a gene from *Burkholderia* (*Pseudomonas*) *cepacia* encoding an outer membrane lipoprotein involved in multiple antibiotic resistance**. *Antimicrob Agents Chemother* 1996, **40**(2):307-313.

6. Nair BM, Cheung KJ, Jr., Griffith A, Burns JL: **Salicylate induces an antibiotic efflux pump in *Burkholderia cepacia* complex genomovar III (*B. cenocepacia*)**. *J Clin Invest* 2004, **113**(3):464-473.

7. Guglierame P, Pasca MR, De Rossi E, Buroni S, Arrigo P, Manina G, Riccardi G: **Efflux pump genes of the resistance-nodulation-division family in *Burkholderia cenocepacia* genome**. *BMC Microbiol* 2006, **6**:66.

8. Kim J, Kim JG, Kang Y, Jang JY, Jog GJ, Lim JY, Kim S, Suga H, Nagamatsu T, Hwang I: **Quorum sensing and the LysR-type transcriptional activator ToxR regulate toxoflavin biosynthesis and transport in *Burkholderia glumae***. *Mol Microbiol* 2004, **54**(4):921-934.

9. Moore RA, DeShazer D, Reckseidler S, Weissman A, Woods DE: **Efflux-mediated aminoglycoside and macrolide resistance in *Burkholderia pseudomallei***. *Antimicrob Agents Chemother* 1999, **43**(3):465-470.

10. Chan YY, Tan TM, Ong YM, Chua KL: **BpeAB-OprB, a multidrug efflux pump in *Burkholderia pseudomallei***. *Antimicrob Agents Chemother* 2004, **48**(4):1128-1135.

11. Chan YY, Chua KL: **The *Burkholderia pseudomallei* BpeAB-OprB efflux pump: expression and impact on quorum sensing and virulence**. *J Bacteriol* 2005, **187**(14):4707-4719.

12. Chan YY, Bian HS, Tan TM, Mattmann ME, Geske GD, Igarashi J, Hatano T, Suga H, Blackwell HE, Chua KL: **Control of quorum sensing by a *Burkholderia pseudomallei* multidrug efflux pump**. *J Bacteriol* 2007, **189**(11):4320-4324.

13. Kumar A, Chua KL, Schweizer HP: **Method for regulated expression of single-copy efflux pump genes in a surrogate *Pseudomonas aeruginosa* strain: identification of the BpeEF-OprC chloramphenicol and trimethoprim efflux pump of *Burkholderia pseudomallei* 1026b**. *Antimicrob Agents Chemother* 2006, **50**(10):3460-3463.

14. Lin J, Cagliero C, Guo B, Barton YW, Maurel MC, Payot S, Zhang Q: **Bile salts modulate expression of the CmeABC multidrug efflux pump in *Campylobacter jejuni***. *J Bacteriol* 2005, **187**(21):7417-7424.

15. Masi M, Pages JM, Villard C, Pradel E: **The eefABC multidrug efflux pump operon is repressed by H-NS in *Enterobacter aerogenes***. *J Bacteriol* 2005, **187**(11):3894-3897.

16. Keeney D, Ruzin A, Bradford PA: **RamA, a transcriptional regulator, and AcrAB, an RND-type efflux pump, are associated with decreased susceptibility to tigecycline in *Enterobacter cloacae***. *Microb Drug Resist* 2007, **13**(1):1-6.

17. Elkins CA, Nikaido H: **Substrate specificity of the RND-type multidrug efflux pumps AcrB and AcrD of *Escherichia coli* is determined predominantly by two large periplasmic loops**. *J Bacteriol* 2002, **184**(23):6490-6498.

18. Rosenberg EY, Ma D, Nikaido H: **AcrD of *Escherichia coli* is an aminoglycoside efflux pump**. *J Bacteriol* 2000, **182**(6):1754-1756.

19. Baranova N, Nikaido H: **The baeSR two-component regulatory system activates transcription of the yegMNOB (mdtABCD) transporter gene cluster in *Escherichia coli* and increases its resistance to novobiocin and deoxycholate**. *J Bacteriol* 2002, **184**(15):4168-4176.

20. Nagakubo S, Nishino K, Hirata T, Yamaguchi A: **The putative response regulator BaeR stimulates multidrug resistance of *Escherichia coli* via a novel multidrug exporter system, MdtABC**. *J Bacteriol* 2002, **184**(15):4161-4167.

21. Nishino K, Yamaguchi A: **EvgA of the two-component signal transduction system modulates production of the yhiUV multidrug transporter in *Escherichia coli***. *J Bacteriol* 2002, **184**(8):2319-2323.

22. Franke S, Grass G, Rensing C, Nies DH: **Molecular analysis of the copper-transporting efflux system CusCFBA of *Escherichia coli***. *J Bacteriol* 2003, **185**(13):3804-3812.

23. Bina XR, Lavine CL, Miller MA, Bina JE: **The AcrAB RND efflux system from the live vaccine strain of *Francisella tularensis* is a multiple drug efflux system that is required for virulence in mice**. *FEMS Microbiol Lett* 2008, **279**(2):226-233.

24. Sanchez L, Pan W, Vinas M, Nikaido H: **The acrAB homolog of *Haemophilus influenzae* codes for a functional multidrug efflux pump**. *J Bacteriol* 1997, **179**(21):6855-6857.

25. Kamal N, Rouquette-Loughlin C, Shafer WM: **The TolC-like protein of *Neisseria meningitidis* is required for extracellular production of the repeats-in-toxin toxin FrpC but not for resistance to antimicrobials recognized by the Mtr efflux pump system**. *Infect Immun* 2007, **75**(12):6008-6012.

26. Hagman KE, Lucas CE, Balthazar JT, Snyder L, Nilles M, Judd RC, Shafer WM: **The MtrD protein of *Neisseria gonorrhoeae* is a member of the resistance/nodulation/division protein family constituting part of an efflux system**. *Microbiology* 1997, **143 ( Pt 7)**:2117-2125.

27. Ikeda T, Yoshimura F: **A resistance-nodulation-cell division family xenobiotic efflux pump in an obligate anaerobe, *Porphyromonas gingivalis***. *Antimicrob Agents Chemother* 2002, **46**(10):3257-3260.

28. Visalli MA, Murphy E, Projan SJ, Bradford PA: **AcrAB multidrug efflux pump is associated with reduced levels of susceptibility to tigecycline (GAR-936) in *Proteus mirabilis***. *Antimicrob Agents Chemother* 2003, **47**(2):665-669.

29. Li XZ, Nikaido H, Poole K: **Role of mexA-mexB-oprM in antibiotic efflux in *Pseudomonas aeruginosa***. *Antimicrob Agents Chemother* 1995, **39**(9):1948-1953.

30. Schweizer HP: **Efflux as a mechanism of resistance to antimicrobials in *Pseudomonas aeruginosa* and related bacteria: unanswered questions**. *Genet Mol Res* 2003, **2**(1):48-62.

31. Poole K, Gotoh N, Tsujimoto H, Zhao Q, Wada A, Yamasaki T, Neshat S, Yamagishi J, Li XZ, Nishino T: **Overexpression of the mexC-mexD-oprJ efflux operon in nfxB-type multidrug-resistant strains of *Pseudomonas aeruginosa***. *Mol Microbiol* 1996, **21**(4):713-724.

32. Mao W, Warren MS, Black DS, Satou T, Murata T, Nishino T, Gotoh N, Lomovskaya O: **On the mechanism of substrate specificity by resistance nodulation division (RND)-type multidrug resistance pumps: the large periplasmic loops of MexD from *Pseudomonas aeruginosa* are involved in substrate recognition**. *Mol Microbiol* 2002, **46**(3):889-901.

33. Kohler T, Epp SF, Curty LK, Pechere JC: **Characterization of MexT, the regulator of the MexE-MexF-OprN multidrug efflux system of *Pseudomonas aeruginosa***. *J Bacteriol* 1999, **181**(20):6300-6305.

34. Poole K: **Bacterial multidrug efflux pumps serve other functions**. *Microbe* 2008, **3**(4):179-185.

35. Aendekerk S, Ghysels B, Cornelis P, Baysse C: **Characterization of a new efflux pump, MexGHI-OpmD, from *Pseudomonas aeruginosa* that confers resistance to vanadium**. *Microbiology* 2002, **148**(Pt 8):2371-2381.

36. Sekiya H, Mima T, Morita Y, Kuroda T, Mizushima T, Tsuchiya T: **Functional cloning and characterization of a multidrug efflux pump, mexHI-opmD, from a *Pseudomonas aeruginosa* mutant**. *Antimicrob Agents Chemother* 2003, **47**(9):2990-2992.

37. Mima T, Sekiya H, Mizushima T, Kuroda T, Tsuchiya T: **Gene cloning and properties of the RND-type multidrug efflux pumps MexPQ-OpmE and MexMN-OprM from *Pseudomonas aeruginosa***. *Microbiol Immunol* 2005, **49**(11):999-1002.

38. Chuanchuen R, Narasaki CT, Schweizer HP: **The MexJK efflux pump of *Pseudomonas aeruginosa* requires OprM for antibiotic efflux but not for efflux of triclosan**. *J Bacteriol* 2002, **184**(18):5036-5044.

39. Li Y, Mima T, Komori Y, Morita Y, Kuroda T, Mizushima T, Tsuchiya T: **A new member of the tripartite multidrug efflux pumps, MexVW-OprM, in *Pseudomonas aeruginosa****. J Antimicrob Chemother* 2003, **52**(4):572-575.

40. Aires JR, Kohler T, Nikaido H, Plesiat P: **Involvement of an active efflux system in the natural resistance of *Pseudomonas aeruginosa* to aminoglycosides**. *Antimicrob Agents Chemother* 1999, **43**(11):2624-2628.

41. Jeannot K, Sobel ML, El Garch F, Poole K, Plesiat P: **Induction of the MexXY efflux pump in *Pseudomonas aeruginosa* is dependent on drug-ribosome interaction**. *J Bacteriol* 2005, **187**(15):5341-5346.

42. Hassan MT, van der Lelie D, Springael D, Romling U, Ahmed N, Mergeay M: **Identification of a gene cluster, czr, involved in cadmium and zinc resistance in *Pseudomonas aeruginosa***. *Gene* 1999, **238**(2):417-425.

43. Caille O, Rossier C, Perron K: **A copper-activated two-component system interacts with zinc and imipenem resistance in *Pseudomonas aeruginosa***. *J Bacteriol* 2007, **189**(13):4561-4568.

44. Mima T, Joshi S, Gomez-Escalada M, Schweizer HP: **Identification and characterization of TriABC-OpmH, a triclosan efflux pump of *Pseudomonas aeruginosa* requiring two membrane fusion proteins**. *J Bacteriol* 2007, **189**(21):7600-7609.

45. Hearn EM, Dennis JJ, Gray MR, Foght JM: **Identification and characterization of the emhABC efflux system for polycyclic aromatic hydrocarbons in *Pseudomonas fluorescens* cLP6a**. *J Bacteriol* 2003, **185**(21):6233-6240.

46. Feng SF, Rossbach,S: **A locus involved in metal homeostasis in *Pseudomonas fluorescens* encodes a proton/cation antiporter of the RND family and a two-component system**. *Unpublished*.

47. Krell T, Teran W, Mayorga OL, Rivas G, Jimenez M, Daniels C, Molina-Henares AJ, Martinez-Bueno M, Gallegos MT, Ramos JL: **Optimization of the palindromic order of the TtgR operator enhances binding cooperativity**. *J Mol Biol* 2007, **369**(5):1188-1199.

48. Duque E, Segura A, Mosqueda G, Ramos JL: **Global and cognate regulators control the expression of the organic solvent efflux pumps TtgABC and TtgDEF of *Pseudomonas putida***. *Mol Microbiol* 2001, **39**(4):1100-1106.

49. Teran W, Krell T, Ramos JL, Gallegos MT: **Effector-repressor interactions, binding of a single effector molecule to the operator-bound TtgR homodimer mediates derepression**. *J Biol Chem* 2006, **281**(11):7102-7109.

50. Rojas A, Duque E, Mosqueda G, Golden G, Hurtado A, Ramos JL, Segura A: **Three efflux pumps are required to provide efficient tolerance to toluene in *Pseudomonas putida* DOT-T1E**. *J Bacteriol* 2001, **183**(13):3967-3973.

51. Leedjarv A, Ivask A, Virta M: **Interplay of different transporters in the mediation of divalent heavy metal resistance in *Pseudomonas putida* KT2440**. *J Bacteriol* 2008, **190**(8):2680-2689.

52. Kieboom J, Dennis JJ, de Bont JA, Zylstra GJ: **Identification and molecular characterization of an efflux pump involved in *Pseudomonas putida* S12 solvent tolerance**. *J Biol Chem* 1998, **273**(1):85-91.

53. Kieboom J, de Bont J: **Identification and molecular characterization of an efflux system involved in *Pseudomonas putida* S12 multidrug resistance**. *Microbiology* 2001, **147**(Pt 1):43-51.

54. Stoitsova SO, Braun Y, Ullrich MS, Weingart H: **Characterization of the RND-type multidrug efflux pump MexAB-OprM of the plant pathogen *Pseudomonas syringae***. *Appl Environ Microbiol* 2008, **74**(11):3387-3393.

55. Liesegang H, Lemke K, Siddiqui RA, Schlegel HG: **Characterization of the inducible nickel and cobalt resistance determinant cnr from pMOL28 of *Alcaligenes eutrophus* CH34**. *J Bacteriol* 1993, **175**(3):767-778.

56. Schmidt T, Schlegel HG: **Combined nickel-cobalt-cadmium resistance encoded by the ncc locus of *Alcaligenes xylosoxidans* 31A**. *J Bacteriol* 1994, **176**(22):7045-7054.

57. Pontel LB, Audero ME, Espariz M, Checa SK, Soncini FC: **GolS controls the response to gold by the hierarchical induction of *Salmonella*-specific genes that include a CBA efflux-coding operon**. *Mol Microbiol* 2007, **66**(3):814-825.

58. Kumar A, Worobec EA: **Cloning, sequencing, and characterization of the SdeAB multidrug efflux pump of *Serratia marcescens***. *Antimicrob Agents Chemother* 2005, **49**(4):1495-1501.

59. Chen J, Kuroda T, Huda MN, Mizushima T, Tsuchiya T: **An RND-type multidrug efflux pump SdeXY from *Serratia marcescens***. *J Antimicrob Chemother* 2003, **52**(2):176-179.

60. Li XZ, Zhang L, Poole K: **SmeC, an outer membrane multidrug efflux protein of *Stenotrophomonas maltophilia****.* *Antimicrob Agents Chemother* 2002, **46**(2):333-343.

61. Alonso A, Martinez JL: **Cloning and characterization of SmeDEF, a novel multidrug efflux pump from *Stenotrophomonas maltophilia***. *Antimicrob Agents Chemother* 2000, **44**(11):3079-3086.

62. Zhang L, Li XZ, Poole K: **SmeDEF multidrug efflux pump contributes to intrinsic multidrug resistance in *Stenotrophomonas maltophilia***. *Antimicrob Agents Chemother* 2001, **45**(12):3497-3503.

63. Crossman LC, Gould VC, Dow JM, Vernikos GS, Okazaki A, Sebaihia M, Saunders D, Arrowsmith C, Carver T, Peters N *et al*: **The complete genome, comparative and functional analysis of *Stenotrophomonas maltophilia* reveals an organism heavily shielded by drug resistance determinants**. *Genome Biol* 2008, **9**(4):R74.

64. Bina XR, Provenzano D, Nguyen N, Bina JE: ***Vibrio cholerae* RND family efflux systems are required for antimicrobial resistance, optimal virulence factor production, and colonization of the infant mouse small intestine**. *Infect Immun* 2008, **76**(8):3595-3605.

65. Matsuo T, Hayashi K, Morita Y, Koterasawa M, Ogawa W, Mizushima T, Tsuchiya T, Kuroda T: **VmeAB, an RND-type multidrug efflux transporter in *Vibrio parahaemolyticus***. *Microbiology* 2007, **153**(Pt 12):4129-4137.
