# Supplementary material for: Exploring the HME and HAE1 efflux systems in the genus Burkholderia
Source: BMC Evol Biol. 2010 Jun 3;10:164. doi: 10.1186/1471-2148-10-164 (PMC2891726; doi:10.1186/1471-2148-10-164)
Supplement: Additional File 4 — Relationship between RND proteins and lifestyle and pathogenicity. Relationship between RND proteins and lifestyle and pathogenicity [file 1471-2148-10-164-S4.PPT]

## Slide 1
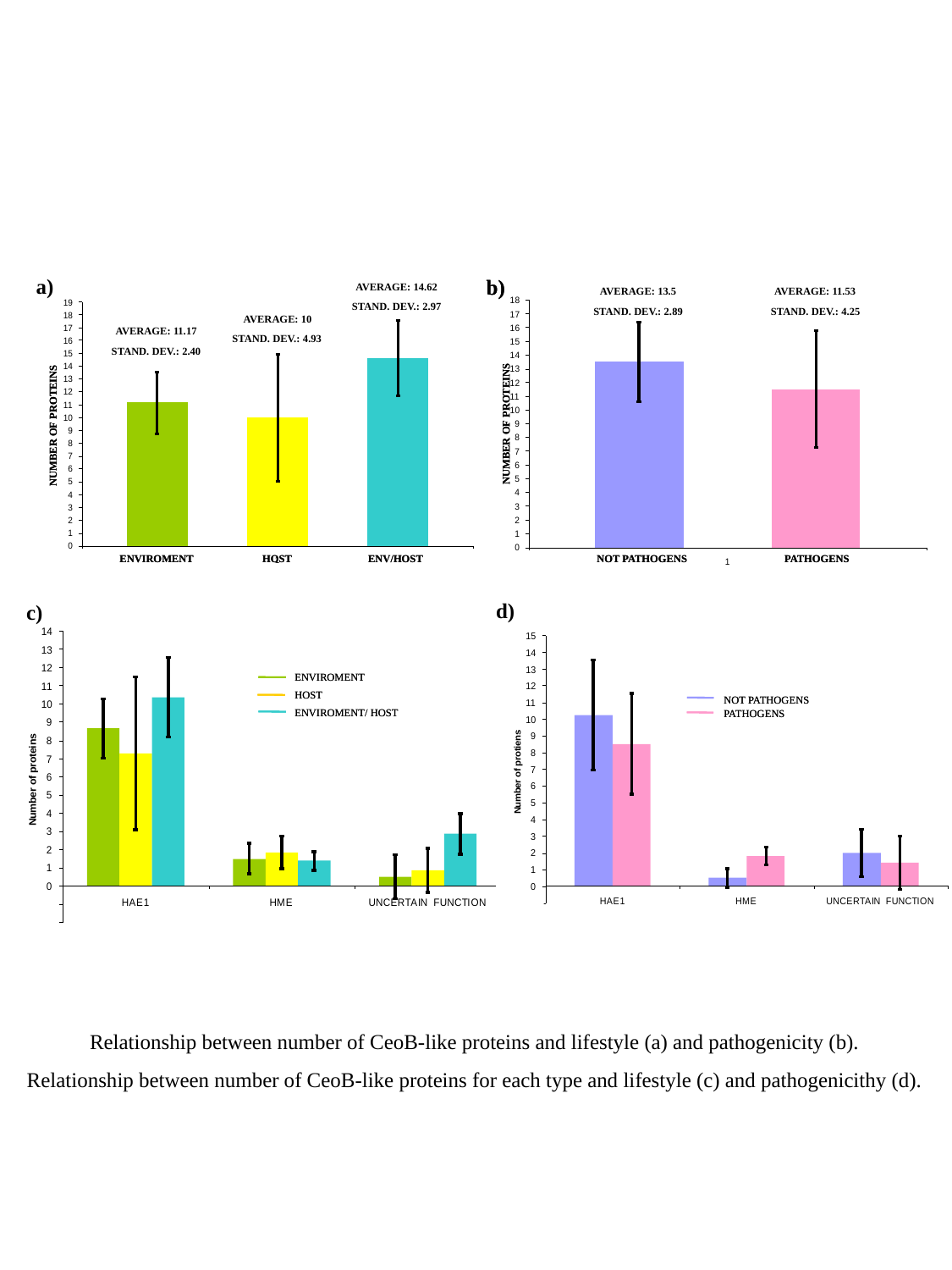

a)
AVERAGE: 14.62
STAND. DEV.: 2.97
AVERAGE: 10
STAND. DEV.: 4.93
AVERAGE: 11.17
STAND. DEV.: 2.40
NUMBER OF PROTEINS
NUMBER OF PROTEINS
ENVIROMENT
ENVIROMENT
HOST
HOST
ENV/HOST
ENV/HOST
b)
b)
AVERAGE: 13.5
STAND. DEV.: 2.89
AVERAGE: 11.53
STAND. DEV.: 4.25
NUMBER OF PROTEINS
NUMBER OF PROTEINS
NOT PATHOGENS
NOT PATHOGENS
PATHOGENS
PATHOGENS
d)
NOT PATHOGENS
NOT PATHOGENS
PATHOGENS
PATHOGENS
c)
ENVIROMENT
ENVIROMENT
HOST
HOST
ENVIROMENT/ HOST
ENVIROMENT/ HOST
Relationship between number of CeoB-like proteins and lifestyle (a) and pathogenicity (b).
Relationship between number of CeoB-like proteins for each type and lifestyle (c) and pathogenicithy (d).
